# Pathological characteristics of axons and alterations of proteomic and lipidomic profiles in midbrain dopaminergic neurodegeneration induced by WDR45-deficiency

**DOI:** 10.1186/s13024-024-00746-4

**Published:** 2024-08-26

**Authors:** Panpan Wang, Yaping Shao, Murad Al-Nusaif, Jun Zhang, Huijia Yang, Yuting Yang, Kunhyok Kim, Song Li, Cong Liu, Huaibin Cai, Weidong Le

**Affiliations:** 1 Liaoning Provincial Key Laboratory for Research On the Pathogenic Mechanisms of Neurological Diseases, The First Affiliated Hospital, Dalian Medical University, Dalian, 116021 China; 2 Interdisciplinary Research Center On Biology and Chemistry, Shanghai Institute of Organic Chemistry, Chinese Academy of Sciences, Shanghai, China; 3 Transgenic Section, Laboratory of Neurogenetics, National Institute on Aging, National Institutes of Health, Bethesda, MD 20892 USA; 4 Institute of Neurology, Sichuan Academy of Medical Science, Sichuan Provincial Hospital, Chengdu, 610072 China

**Keywords:** WDR45, Axonal degeneration, Autophagy, Tubular ER, Phospholipid metabolism, Lpcat1

## Abstract

**Background:**

Although *WD repeat domain 45 (WDR45)* mutations have been linked to $\upbeta$-propeller protein-associated neurodegeneration (BPAN), the precise molecular and cellular mechanisms behind this disease remain elusive. This study aims to shed light on the impacts of WDR45-deficiency on neurodegeneration, specifically axonal degeneration, within the midbrain dopaminergic (DAergic) system. We hope to better understand the disease process by examining pathological and molecular alterations, especially within the DAergic system.

**Methods:**

To investigate the impacts of WDR45 dysfunction on mouse behaviors and DAergic neurons, we developed a mouse model in which *WDR45* was conditionally knocked out in the midbrain DAergic neurons (*WDR45*^*cKO*^). Through a longitudinal study, we assessed alterations in the mouse behaviors using open field, rotarod, Y-maze, and 3-chamber social approach tests. We utilized a combination of immunofluorescence staining and transmission electron microscopy to examine the pathological changes in DAergic neuron soma and axons. Additionally, we performed proteomic and lipidomic analyses of the striatum from young and aged mice to identify the molecules and processes potentially involved in the striatal pathology during aging. Further more, primary midbrain neuronal culture was employed to explore the molecular mechanisms leading to axonal degeneration.

**Results:**

Our study of *WDR45*^*cKO*^ mice revealed a range of deficits, including impaired motor function, emotional instability, and memory loss, coinciding with the profound reduction of midbrain DAergic neurons. The neuronal loss, we observed massive axonal enlargements in the dorsal and ventral striatum. These enlargements were characterized by the accumulation of extensively fragmented tubular endoplasmic reticulum (ER), a hallmark of axonal degeneration. Proteomic analysis of the striatum showed that the differentially expressed proteins were enriched in metabolic processes. The carbohydrate metabolic and protein catabolic processes appeared earlier, and amino acid, lipid, and tricarboxylic acid metabolisms were increased during aging. Of note, we observed a tremendous increase in the expression of lysophosphatidylcholine acyltransferase 1 (Lpcat1) that regulates phospholipid metabolism, specifically in the conversion of lysophosphatidylcholine (LPC) to phosphatidylcholine (PC) in the presence of acyl-CoA. The lipidomic results consistently suggested that differential lipids were concentrated on PC and LPC. Axonal degeneration was effectively ameliorated by interfering Lpcat1 expression in primary cultured WDR45-deficient DAergic neurons, proving that Lpcat1 and its regulated lipid metabolism, especially PC and LPC metabolism, participate in controlling the axonal degeneration induced by WDR45 deficits.

**Conclusions:**

In this study, we uncovered the molecular mechanisms underlying the contribution of WDR45 deficiency to axonal degeneration, which involves complex relationships between phospholipid metabolism, autophagy, and tubular ER. These findings greatly advance our understanding of the fundamental molecular mechanisms driving axonal degeneration and may provide a foundation for developing novel mechanistically based therapeutic interventions for BPAN and other neurodegenerative diseases.

**Supplementary Information:**

The online version contains supplementary material available at 10.1186/s13024-024-00746-4.

## Background

*Tryptophan-aspartic acid (WD) repeat domain 45 (WDR45)*, encoding a superfamily of proteins characterized by repeating units with a conserved core of approximately 40 amino acids, is located on the X-chromosome [1]. WD-repeat proteins have a highly symmetrical β-propeller tertiary structure that enables them to regulate the assembly of multiprotein complexes by providing a stable anchoring platform [2]. Based on this structural property, it seems that the WDR45 protein plays key roles in many biological processes, including autophagy, an autophagosome-lysosome-mediated degradation system [3], transduction, and vesicular trafficking [4, 5]. De novo mutations in the *WDR45* gene were recently identified in β-propeller protein-associated neurodegeneration (BPAN) disease [6, 7]. BPAN is characterized as a subtype of neurodegeneration with brain iron accumulation, and clinically, patients with *WDR45* mutations have a biphasic disease course that begins with global developmental delay in infancy or early childhood, and subsequent progressive cognitive decline, dementia, dystonia, and Parkinsonism in adolescence or early adulthood [8–10]. Moreover, brain imaging from BPAN patients with mutated *WDR45* displays generalized brain atrophy and bilateral mineralization of the substantia nigra (SN) and globus pallidus [8, 11]. In addition to BPAN disorder, *WDR45* mutations have been linked to pancreatic cancer, kidney cancer, and Rett syndrome [12, 13]. Rett syndrome is a neurodevelopmental disorder characterized by the loss of purposeful hand skills, language regression, stereotypic hand movements, and gait abnormalities. Some BPAN patients have features like Rett syndrome [13, 14].

The endoplasmic reticulum (ER) is an interconnected network of tubular and planar membranes that supports synthesizing and exporting proteins, carbohydrates, and lipids [15]. ER is also the primary site for phospholipid synthesis, particularly phosphatidylcholine (PC) synthesis, and accounts for more than 60% of phospholipid mass [16]. The phospholipid content and composition of the ER are dynamic for the regulated export to other membrane organelles and secretion of lipids to the extracellular environment. Lipid biosynthesis needs to match these export demands to maintain ER integrity and the membrane requirements of distal organelles. In the neuron, the extended ER network stretched out to axons, where it provides the lipids to maintain the functions of the axonal organelles. Once the lipid metabolism balance is disrupted, the maintenance and integrity of axonal morphology are inevitably affected [17]. The regulation of lipid metabolic enzymes could also induce membrane deformation because many of them, like phosphate cytidylyltransferase 1A, choline [18], and phospholipase A2 group IVA, contain membrane curvature-inducing domains [19].

Axonal ER comprises tubular ER and forms a physically continuous network of interconnected tubular structures, participating in axonal morphology, transport, and material metabolism, suggesting its potential role in neurodegeneration [20]. The tubular ER structure is determined by ER-shaping proteins, such as reticulons (RTNs), receptor accessory proteins (REEPs), and the atlastins (ATLs) family. Mutations in these ER-shaping proteins can cause hereditary spastic paraplegia [20]. The accumulation of tubular ER is primarily specific to axons [21]. Recent studies have revealed the physiological roles of tubular ER in neurodegenerative disorders [20, 22, 23]. Autophagy gene 5 (ATG5) deletion leads to tubular ER accumulation in axons of hippocampal neurons [21], demonstrating that autophagy participates in the axonal tubular ER degradation.

The tubular ER dynamics are closely linked to phospholipid synthesis. Cytidine diphosphate-triacylglycerol (CDP-DAG) synthase 1, a crucial enzyme of PC synthesis through the CDP-DAG pathway, is uniformly localized to the tubular ER and nuclear envelope. Phosphatidylinositol synthase (PIS), the rate-limiting enzyme for phosphatidylinositol synthesis, also localizes to the tubular ER network. Rab10, an ER-specific Rab GTPase, regulates ER tubule dynamics and tubular ER morphology by controlling ER tubule extension and fusion at the leading edge of ER dynamics. Further study suggests that these dynamics could be coupled to phospholipid synthesis since the Rab10 domain is highly enriched with at least two ER enzymes that regulate phospholipid synthesis, PIS, and choline/ethanolamine phosphotransferase 1 [24].

Using a line of newly developed *WDR45* conditional knockout (cKO) mice, our study shows a correlation between axonal degeneration, the accumulation of aberrant tubular ER, and phospholipid metabolism in WDR45-deficient midbrain DAergic neurons. These findings suggest phospholipid metabolism may play a role in WDR45 deficient-induced axonal degeneration. By uncovering these connections, our study provides new insights into the molecular mechanisms underlying WDR45-deficiency-induced neurodegeneration and highlights the potential targets for developing therapeutic interventions to prevent or reverse this process.

## Materials and methods

### Generation of the conditional knockout *WDR45* mouse model

Heterozygous *WDR45*^*Flox/wt*^ mice were generated by ViewSolid Biotech Co., Ltd. (Beijing, China). Briefly, CRISPR/Cas9 technology replaced the genomic DNA fragment from intron 2 and 4 of the *WDR45* gene with a donor DNA fragment containing LoxP-flanked exon 2 to 4 *WDR45*. Based on Cas9/gRNA activity screen and target location, high-activity gRNAs (target DNA sequence: GCACAAACACCAAGCATGGGG; ACACAGTGCTATTGGGGCTGG) were selected for microinjection into C57BL/6 J fertilized eggs to produce conditional gene knockout mice.

To achieve a mouse model that conditionally knocked out *WDR45* in the DAergic system, we bred *DAT*^*CreERT2*^ mice carrying inducible Cre recombinase under the DAT promoter with heterozygous *WDR45*^*Flox/wt*^ mice to obtain *WDR45*^*Flox/Flox*^*/DAT*^*CreERT2*^ mice. The *DAT*^*CreERT*2^ mouse was kindly gifted by the Günther Schütz group and was generated by recombining a construct containing a modified Cre recombinase fused to a modified ligand-binding domain of the estrogen receptor into a bacterial artificial chromosome containing the gene encoding *DAT* [25, 26].

To achieve the conditional knockout of *WDR45* in the mature DAergic system, tamoxifen (TAM, T-5648; Sigma‒Aldrich) was employed to treat mice. TAM has dissolved in a corn oil/ethanol (S-5007; Sigma‒Aldrich) mixture with a ratio of 10:1 at a final concentration of 10 mg/mL. A fresh mixture was prepared by shaking overnight to dissolve TAM completely at 4 °C and then stored at -20 °C for 2 weeks. Eight-week-old *WDR45*^*Flox/Flox*^* (WDR45*^*cWT*^*) and WDR45*^*Flox/Flox*^*/DAT*^*CreERT2*^ (*WDR45*^*cKO*^) mice were both injected intraperitoneally with 1 mg TAM twice daily (total 2 mg/day, approximately 25 mg/kg body weight) for 5 consecutive days. Behavioral tests were performed at the ages of 6–8 months, 11–13 months, and 17–19 months. *WDR45*^*cWT*^ and *WDR45*^*cKO*^ mice were sacrificed at the scheduled time (Fig. S1a).

*DAT*^*CreERT2*^ transgenic mice were identified by PCR screening (2 × EasyTaq PCR SuperMix, Transgene Biotech) of tail DNA using an antisense primer, CAG ACC AGG CCA GGT ATC TCT, and a sense primer, AGA ACC TGA TGG ACA TGT TCA GG, of which the transgene band size is 700 bp. Floxed *WDR45* knock-in mice were identified using CCACAGTAAGGCACAGTT and GTACAGACCAGGCAAGTG. The PCR product size of the wild-type allele was 179 bp, and the knock-in Flox allele was 213 bp.

All mice were maintained under SPF conditions (temperature, 22 ± 2 °C; air exchange per 20 min; 12 h/12 h light/dark cycle with the light on at 6:00 AM) with free access to food and water. Animal care and procedures were carried out per the Laboratory Animal Care Guidelines approved by the Institutional Animal Care Committee at Dalian Medical University. The protocol was approved by the Institutional Animal Care Committee at Dalian Medical University.

### Behavioral tests

#### Locomotor activity

To examine their locomotor activity, *WDR45*^*cWT*^ and *WDR45*^*cKO*^ mice were placed in a locomotor activity monitor (25 × 25 × 30 cm, Med Associates Inc., St. Albans, USA) equipped with a computer-controlled photocell. The activity was automatically tracked and recorded for 10 min for the total distance traveled and stereotypic time. The assessment was conducted on the scheduled date between 13:00 and 16:00 at the ages of 6–8 months, 11–13 months, and 17–19 months. Both male and female mice were employed to test their locomotor activity. The same mice were tested for locomotor activity at these three age stages. Test performers were blinded to the genotypes of the mice.

#### Rotarod test

As described previously, mice were trained on the IITC Rotarod (IITC Life Science, Woodland Hills, CA) at 5 r/minute, twice per day (at 1-h intervals) for 3 consecutive days, and then on the fourth day, they were tested on the rotating rod with speed auto accelerating from 4 to 40 r/minute over a period of 5 min. The time spent on the rotating rod for each mouse was recorded across three trials at 1-h intervals. The behavioral assessment was performed at ages 6–8 months, 11–13 months, and 17–19 months. Both male and female mice were employed for the rotarod test. The same mice were employed for the rotarod test at these three age stages. Test performers were blinded to the genotypes of the mice.

#### Y-maze test

The Y-maze test apparatus (Beijing Zhongshidichuang Science and Technology Development Col., Ltd, Beijing, China) was implemented on a white background with three arms (labeled a, b, and c arms) that extended from a central platform at a 120° angle. Each mouse was placed in the center and allowed to freely explore the maze for 6 min. The sequence and the total number of arms that the mouse entered were recorded using the observer. An arm entry was successful when the mouse's whole body was within the arm. Both male and female mice were employed to the Y-maze test. The same mice were employed for the Y-maze test at these three age stages. Test performers were blinded to the genotypes of the mice.

#### Three-chamber social approach test

The three-chamber social approach test was performed as described previously [4]. Briefly, a conspecific mouse of the same sex was placed in a wire-framed steel cage within either the left or right chamber (named novel, the left steel named other) and the subject mouse was allowed to move freely among the three chambers for 5 min. A second novel mouse (matched for age and sex) was placed in the remaining wired framed steel cage (named novel, the previous one named familiar), and the subject mouse could move freely for an additional 5 min. The relative exploration time of the mouse to enter each zone was measured. Male mice were employed for Three-chamber social approach test at the ages of 11–13 months and 17–19 months, and the same mice were tested at these two age stages. Test performers were blinded to the genotypes of the mice.

### Primary midbrain neuronal culture and transfection

Mouse primary midbrain neuronal cultures were prepared from newborn *WDR45*^*Flox/Flox*^*/DAT*^*CreERT2*^ pups and littermate controls on P0. Briefly, midbrain tissues containing SNc and VTA were dissected and subjected to trypsin digestion (mixed with DMEM/F12 to a final concentration of 0.125 mg/ml, Gibco) for 20 min at 37 °C. The digested tissue was carefully triturated into single cells using pipette tips. The cells were then centrifuged at 250 × g for 5 min and resuspended in warm DMEM/F12 containing 10% fetal bovine serum (FBS, Invitrogen) medium. After filtrating with 70 μm sieve, the dissociated cells were seeded in 12-mm round coverslips precoated with poly-D-lysine and laminin (BD Bioscience) in a 24-well plate and maintained at 37 °C in the 95% O_2_- and 5% CO_2_-humidified incubator. 12 h after seeding, the cultures were switched to the neurobasal medium containing 1 × B27 supplement (the optimized serum-free supplement used to support the growth and viability of neurons, 100 × stock, Invitrogen) and 1 × GlutaMax (100 × stock, Invitrogen). After culturing for 7 days in vitro (DIV), lentivirus (LV)-shLpcat1 (primer: 5’- GGAAGACAGTGGAGGAGATCA-3’), LV-NC (primer: 5’-TTCTCCGAACGTGTCACGT-3’) (MOI = 3, GenePharma), or pHBLV-m-Lpcat1-3Flag (gene sequence: NM_145376.6; MOI = 3, HANBIO) were transfected into neuron cultures for 24 h according to the manufacturer’s protocol. 1 μM 4-Hydroxytamoxifen (4-OHT) (Sigma-Aldrich) was added to induce CRE recombinase activity at DIV 8 and culture for 5 consecutive days. From DIV 2, half of the culture medium was replaced every 3 days.

### Immunofluorescence (IFC) staining and image analysis

Mice were anesthetized with ketamine and perfused transcardially with 40 mL PBS and then 60 mL 4% paraformaldehyde (PFA). After dehydrating 30% sucrose for 72 h, the brain tissues were cut into 40 μm coronal sections using a Leica cryostat (CM-1950S, Leica, Germany). The slides were incubated with an IFC blocking buffer (10% normal goat serum, 1% bovine serum albumin, 0.3% Triton X-100, PBS solution) for 2 h at room temperature and were then incubated with the primary antibodies overnight at 4 °C (a complete list of primary antibodies information in Table 1). For ubiquitin (Ub) staining, the sections were subjected to antigen repair using citrate buffer (pH 6.0). The stained sections were visualized and photographed directly with a laser scanning confocal microscope (A1 confocal, Nikon Instruments (Shanghai) Co., Ltd). The paired images in the figures were collected at the same gain and offset settings.

**Table 1:** Antibodies used in this study

Target	Species	Application	Dilution	Company	Cat. no
TH	Chicken	IFC	1:1000	Millipore	AB9702
TH	Rabbit	IFC	1:1000	Millipore	AB152
WDR45	Rabbit	IFC	1:500	Novus	NBP3-04699
LC3β	Rabbit	IFC	1:400	Novus	NB100-2220
LC3β	Mouse	IFC	1:500	CST	83560 s
P62	Rabbit	IFC	1:400	Abcam	Ab109012
Lamp1	Rat	IFC	1:500	Abcam	Ab25245
MLKL (phospho S345)	Rabbit	IFC	1:400	Abcam	Ab196436
Ubiquitin	Mouse	IFC	1:200	SANTACRUZE	Sc8017
RTN3	Rabbit	IFC	1:1000	Millipore	ABN1723
REEP2	Rabbit	IFC	1:500	Proteintech	15,684–1-AP
REEP5	Rabbit	IFC	1:500	Proteintech	14,643–1-AP
Phospho-RIP3 (Thr231/Ser232)	Rabbit	IFC	1:400	CST	91,702
KDEL	Rabbit	IFC	1:250	Abcam	Ab176333
RTN4	Mouse	IFC	1:300	SANTA	Sc-271878
Climp-63	Mouse	IFC	1:300	SANTA	Sc-393544
NMNAT3	Mouse	IFC, WB	1:300	SANTA	Sc-390433
Lamp1	Rat	IFC	1:500	Proteintech	65,050–1-Ig
PSD95	Rabbit	IFC	1:500	Synaptic system	N3783
Synapsin1	Rabbit	IFC	1:500	Synaptic system	106,103
Synaptotagmin 1	Mouse	IFC	1:500	Synaptic system	105,011
HOMER1	Rabbit	IFC	1:500	Synaptic system	160,003
Bassoon	Chicken	IFC	1:500	Synaptic system	141,016
DRD1	Rabbit	IFC	1:500	Proteintech	17,934-l-AP
vMAT2	Rabbit	IFC	1:500	Proteintech	20,873-l-AP
Lpcat1	Rabbit	IFC, WB	1:500	Proteintech	16,112-l-AP
DRD2	Rabbit	IFC	1:500	Proteintech	55,084-l-AP
TOM20	Rabbit	IFC	1:400	CST	42406S
BNIP3	Rabbit	IFC	1:250	Abcam	109,362
FIS1	Rabbit	IFC	1:400	Proteintech	10,956–1-AP
MFN1	Rabbit	IFC	1:400	Proteintech	13,798–1-AP
OPA1	Rabbit	IFC	1:400	Proteintech	27,733–1-AP
SEC16A	Rabbit	IFC	1:400	Proteintech	20,025–1-AP
SEC31A	Rabbit	IFC	1:400	CST	13466S
ATL3	Rabbit	IFC	1:200	Proteintech	16,921–1-AP

DAergic neurons in the SNc and VTA were calculated from nine slices per mouse from bregma − 2.80 to − 3.64 mm, we collected one in every three. The outline of the SNc and VTA was determined according to anatomical landmarks. The analysis of IFC staining on the number of the puncta, axon density, and the mean number of enlarged axon terminals was quantified using ImageJ software. After adjusting the threshold and carefully marking the borders of the SN and VTA, only TH-positive cell bodies with a visible nucleus in the blue channel were manually counted by ImageJ (Cell Counter plugin). The total number of TH-positive neurons for the entire SN and VTA in one side of the mouse was estimated by multiplying the counted cell number by three since we collected slices in every three sections. The density of the DAergic axon terminals (fibers) from the same striatal sections was determined as described previously [27]. In brief, z-stack images were acquired (2 µm step size, 35 µm scan range). TH-positive fibers were delineated from the maximal intensity projection (Image J) after adjusting the threshold, noise removal, and binarization, and the density was calculated and expressed as relative density. Enlarged terminals that an area larger than 5 µm^2^ were counted by Image J (analyze particles) after adjusting the threshold and noise removal, then calculated the density of enlargements per 0.045mm^2^ perspective. Evaluate the PSD95, SYN1, SYT1, HOMER1, and BSN expression by using ImageJ for integrated density calculation after adjusting the threshold. For quantification of colocalization of TH-positive axonal enlargements and individual targets, like RTN3, REEP2, and REEP5, we first use Image J to adjust the threshold and noise removal (background subtraction), and then use the coloc2 plugin to analyze whether the two target proteins are colocalized. If so, conduct “analyze particles” to obtain each area value of enlargement that larger than 5 µm^2^. Enlargements with an area larger than 5 µm^2^ were collected by Image J (analyze particles) after adjusting the threshold and noise removal in primary DAergic neurons, and then the ratio of total TH-positive swellings distributed on all axons to the number of axons in each DAergic neuron, that is, the mean density of swellings on each axon, was calculated.

### Quantitative real-time PCR (qRT-PCR)

Mice were decapitated, and the striatum was isolated on ice quickly. Total RNA was extracted using TRIzol reagent (Invitrogen, Carlsbad, CA, United States), and reverse transcription was performed according to the manufacturer’s instructions (638,315, Clontech Laboratories, Inc., A Takara Bio Company, United States). qRT-PCR was performed to determine the expression levels of lipid metabolism-related genes using a proper qRT-PCR kit (a complete list of qRT-PCR primers information in Table 2). The relative gene expression was normalized to *GAPDH* and assessed using the 2^−ΔCT^ method.

**Table 2:** Primers used for qRT-PCR

Primer	5’-F	5’-R
Lpcat1	GGCTCCTGTTCGCTGCTTT	TTCACAGCTACACGGTGGAAG
Snx32	GCTGGAAATGAGAGTAAGCCTT	GGTGTCATGTAGCCAGATGAACT
Abhd4	GGCACAGTTTGGGAGGATTCC	ACTAGGGTCAGTTGGTCGTAG
Etnppl	AGAGGGAGGAACATTCATTGACT	GGCTCGCATTATTTTGATGGGA
MBP	GGCGGTGACAGACTCCAAG	GAAGCTCGTCGGACTCTGAG
APOD	TCACCACAGCCAAAGGACAAA	CGTTCTCCATCAGCGAGTAGT
Tiam2	ACATGGTTGGACTCATGGGAG	TGGTGCCCTTTGAGACTTTACA
Ophn1	ACCCCTGGAAACTTTTCGGAA	TCTGCCTCTAGTAGCTGAGATTC
GAPDH	AGGTCGGTGTGAACGGATTTG	TGTAGACCATGTAGTTGAGGTCA

### Transmission electron microscope (TEM) analysis

Using rodent brain matrice on ice, the midbrain containing SN (bregma: about -2.70 mm to -3.70 mm) and striatum (bregma: about 1.10 mm to 0.02 mm) were dissected accurately, and then the tissues (2 mm x 1 mm x 1 mm) were cut along the edge of the SN reticular region rapidly within 3 min. The tissues were then put into a fixative solution containing 2.5% glutaraldehyde (Servicebio, Wuhan, China) for 2 h fixation at room temperature, followed by transfer to 4 degrees for storage. The tissues were washed three times in PBS before postfixing in 1% osmium acid (diluted with 0.1 M PBS solution) at room temperature for 2 h and were successively dehydrated. After embedding steps, tissues were cut into 80 nm sections using a Leica ultrathin microtome (Leica UC7, Leica, Germany) and stained with 2% uranyl acetate saturated alcohol and lead citrate solution. The stained sections were imaged using TEM (HITACHI, HT7700).

The TEM pictures were analyzed by Image J. In detail, scale and measurements were first set (Analyze-Set Scale/Set Measurements), followed by circling each observed mitochondrion by using a segmented line to calculate the mitochondrial perimeter. Measure the interval of the RER tubule by using a straight line to get the width of the RER tubule. The total numbers of mitochondria, mitochondria with damaged cristae, and RER tubules in each perspective were collected using cell counter (Plugins-analyze). Evaluate PSD95 area and width also by Image J, using a segmented line to circle each observed PSD95 and using a straight line to get its interval, then calculate its area and width. For quantification of the length of tubular ER in the striatum, we first set a scale and then use a segmented line to measure the length of each observed tubular ER and calculate the mean length of collective tubular ER per perspective.

### Proteomic Analysis

#### Sample preparation

The striatal samples were ground into cell powder with liquid nitrogen before being transferred to a 5-mL centrifuge tube. 4 mL lysis buffer (8 M urea, 1% protease inhibitor cocktail) was added to the cell powder, then sonicated three times on ice using a high-intensity ultrasonic processor (Scientz). The remaining debris was removed by centrifugation at 12,000 g at 4 °C for 10 min. The supernatant and the protein concentration were determined with a BCA kit according to the manufacturer’s instructions. For digestion, the protein solution was reduced with 5 mM dithiothreitol for 30 min at 56 °C and alkylated with 11 mM iodoacetamide for 15 min at room temperature in darkness. The protein sample was then diluted by adding 100 mM TEAB to urea concentration less than 2 M. Trypsin was added at 1:50 trypsin-to-protein mass ratio for the first digestion overnight and at 1:100 trypsin-to-protein mass ratio for a second 4 h-digestion. The peptides were desalted by the C18 SPE column.

#### LC–MS/MS-based proteomic analysis

The tryptic peptides were dissolved in solvent A (0.1% formic acid, 2% acetonitrile/in water) and directly loaded onto a reversed-phase analytical column (25-cm length, 75/100 μm i.d.). Peptides were separated with a gradient from 6 to 24% solvent B (0.1% formic acid in acetonitrile) over 70 min, 24% to 35% in 14 min, and climbing to 80% in 3 min, then holding at 80% for the last 3 min at a constant flow rate of 450 nL/min on a nanoElute UHPLC system (Bruker Daltonics). The peptides were subjected to a capillary source followed by the timsTOF Pro (Bruker Daltonics) mass spectrometry. The electrospray voltage applied was 1.60 kV. Precursors and fragments were analyzed at the TOF detector, with an MS/MS scan range from 100 to 1700 m/z. The timsTOF Pro was operated in parallel accumulation serial fragmentation (PASEF) mode. Precursors with charge state 0 to 5 were selected for fragmentation, and 10 PASEF-MS/MS scans were acquired per cycle. The dynamic exclusion was set to 30 s. The MS/MS data were processed using MaxQuant search engine (v.1.6.15.0). Tandem mass spectra were searched against the human SwissProt database (20,422 entries) concatenated with the reverse decoy database. Trypsin/P was specified as a cleavage enzyme allowing up to 2 missing cleavages. The mass tolerance for precursor ions was set as 20 ppm in the first search, and 5 ppm in the main search, and the mass tolerance for fragment ions was set as 0.02 Da. Carbamidomethyl on Cys was specified as a fixed modification, and acetylation on protein N-terminal and oxidation on Met were specified as variable modifications. FDR was adjusted to < 1%.

#### Bioinformatic analysis for proteomics

GO analysis mainly includes three aspects: 1. Cellular component: Refers to the specific component of the cell. 2. Molecular function: Mainly describe the chemical activity of the molecule, such as the catalytic activity or binding activity at the molecular level. 3. Biological process: a series of elements in the body that execute a specific function in order, called the biological process. GO annotation is to annotate and analyze the identified proteins with eggnog-mapper software (v2.0). The software is based on the EggNOG database. The latest version is the 5th edition, covering 5,090 organisms (477 eukaryotes, 4445 representative bacteria, and 168 archaebacteria) and 2502 virus genome-wide coding protein sequences. Extracting the GO ID from the results of each protein note and then classifying the protein according to cellular component, molecular function, and biological process. Fisher’s exact test is used to analyze the significance of GO enrichment of differentially expressed proteins (using the identified protein as the background), and *p* < 0.05 is considered significant.

### Lipidomic analysis

#### UPLC-MS/MS-based lipidomic analysis

For each striatal tissue sample, approximately 10 mg of tissue was weighed and placed into a 2 mL centrifuge tube. Lipids in tissues were extracted using a methyl tert-butyl ether-methanol-H_2_O extraction system, as described in our previous study [28, 29] Non-targeted lipidomics analysis was performed using a hyphenated liquid chromatography-mass spectrometry system equipped with ACQUITY™ ultra-performance liquid chromatography (UPLC) (Waters; Milford, MA, USA) and AB Sciex tripleTOF 5600 plus mass spectrometer (AB Sciex; Framingham, MA, USA). An ACQUITY UPLC BEH C8 (1.7 μm, 2.1 × 100 mm) column was used for chromatographic separation. Peak alignment, peak integration as well as lipid identification based on accurate mass, chromatographic retention, and tandem mass spectrometry (MS/MS) fragmentation patterns were performed using MS-DIAL (Ver.5.1) software. The raw data were normalized to the total intensity of all detected ions in each sample before further statistical analysis.

#### Statistical analysis for lipidomics

Partial least squares-discriminant analysis (PLS-DA) was conducted by SIMCA software (version 13.0.0.0., Umetrics AB, Umea, Sweden). Two-sided independent Student’s *t*-test was applied to determine the DELs between *WDR45*^*cKO*^ mice and WT mice. Heat maps that visualize the changes of lipids in different groups were made by MeV software package (version 4.8.1).

### Statistical analysis

Data are expressed as the means ± SEMs and were analyzed using GraphPad Prism software (version 9.0). Shapiro–Wilk test was used to test normality of the data sets and graphically by QQ plot. Two-way ANOVA followed by Sidak's multiple comparisons test or Tukey's multiple comparisons test was used for analyses in all cases where 2 independent factors were examined (i.e. age and genotype), with unpaired t-test (parametric test was used when the data pass the normality test, if not, non-parametric test was used) used to determine significant differences in cases when no interaction was observed between the independent factors. *p* < 0.05 is considered significant. All experiments were repeated at least three times, and pilot experiments estimated sample sizes. The statistical tests used for each analysis, the sample size and the significance levels were reported in the legend of each figure.

## Results

### Progressive midbrain DAergic neuronal reduction in the SN of *WDR45*^*cKO*^mice

To create a mouse model with selective deletion of *WDR45* in the midbrain DAergic neurons, we used a TAM-inducible *CreERT2/loxp* gene-targeting system (Fig. S1a). *WDR45*-floxed mice were crossed with *DAT*^*CreERT2*^ mice to generate *WDR45*^*Flox/Flox*^*/DAT*^*CreERT2*^ mice. *WDR45*^*Flox/Flox*^ mice were used as controls (W*DR45*^*cKO*^ and *WDR45*^*cWT*^, respectively), and their genotype was confirmed by using conventional PCR analysis (Fig. S1b). When the mice reached 8 weeks of age, we administered intraperitoneal injections of TAM to both *WDR45*^*cWT*^ and *WDR45*^*cKO*^ mice. Tissues were collected 4 months after TAM administration (when the mice were 6 months old), and we used immunofluorescence (IFC) staining to detect WDR45 protein expression in the tyrosine hydroxylase (TH)-labeled DAergic neurons. The results showed a marked decrease in WDR45 expression in the DAergic neurons of *WDR45*^*cKO*^ mice (Fig. S1c, d), indicating the successful deletion of WDR45 in these neurons.

*WDR45*^*cWT*^ and *WDR45*^*cKO*^ mice were subjected to behavioral tests at different ages, including 6–8 months (young), 11–13 months (middle-aged), and 17–19 months (aged). The results showed that the aged *WDR45*^*cKO*^ mice had a significant motor impairment, as indicated by a decrease in the total distance traveled in the open-field test (Fig. S2a) and a notable reduction in stereotypic counts (Fig. S2b), suggesting an increased vulnerability to motor activity impairments with aging. However, no abnormalities were observed in the rotarod test (Fig. S2c). In addition to the locomotion deficits, the aged *WDR45*^*cKO*^ mice also showed poor immediate spatial working memory performance, as evidenced by a decreased spontaneous alteration proportion in the Y maze test (Fig. S2d). Furthermore, the 3-chamber social performance test revealed that the aged *WDR45*^*cKO*^ mice displayed less motivation in novelty (Fig. S2e-h). Moreover, *WDR45*^*cKO*^ mice spent less time in exploration (Fig. S2i, j), suggesting that WDR45 dysfunction in midbrain DAergic neurons may lead to depression-like behavior in aging mice.

**Fig. 1:**
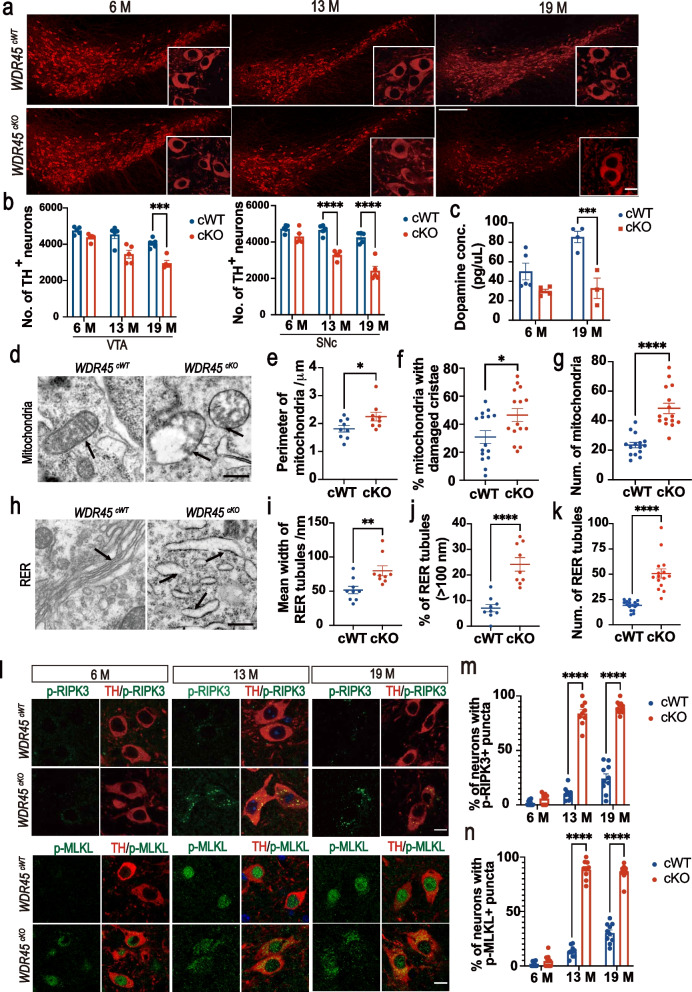
DAergic neuronal reduction in the SN. **a** IFC staining was performed using an antibody against TH (red) in midbrains from young (6–8 months old), middle-aged (11–13 months old), and aged (17–19 months old) *WDR45*^*cWT*^ and *WDR45*^*cKO*^ mice. Scale bar, 250 μm. Scale bar for high-magnification images, 10 μm. **b** Quantifying TH-positive neurons in the VTA and SNc of *WDR45*^*cWT*^ and *WDR45*^*cKO*^ mice (*N* = 5 mice per genotype). **c** The dopamine concentration in the SN region was detected by high-performance liquid chromatography (*N* = 3–5 mice per genotype). **d** Representative TEM images of observed mitochondria in aged *WDR45*^*cWT*^ mice and *WDR45*^*cKO*^ mice. Scale bar, 500 nm. **e** Quantification of the perimeter of mitochondria in DAergic neurons (*N* = 154 mitochondria collectively counted from 9 slices of 3 *WDR45*^*cWT*^ mice and 251 mitochondria from 9 slices of 3 *WDR45*^*cKO*^ mice). **f** The proportion of mitochondria with damaged cristae was quantified (*N* = 15 slices from 3 mice per genotype). **g** The mean number of mitochondria observed in captured images was collected (*N* = 15 slices from 3 mice for each genotype). **h** Representative TEM images of observed RER. Scale bar, 500 nm. **i** The mean width of RER tubules is shown (*N* = 163 RER collectively counted from 9 slices of 3 *WDR45*^*cWT*^ mice and 288 RER from 9 slices of 3 *WDR45*^*cKO*^ mice). **j** The proportion of RER tubules (> 100 nm) was quantified (*N* = 15 slices from 3 mice per genotype). **k** The mean number of RER observed in captured images was collected (*N* = 15 slices from 3 mice for each genotype). **l** Double-label immunofluorescence of p-RIPK3 (Thr 231/Ser232) or p-MLKL (phosphor S345) (green) with TH (red) in the DAergic neurons of young, middle-aged, and aged *WDR45*^*cWT*^ mice and *WDR45*^*cKO*^ mice. Scale bar, 10 μm. **m** The proportion of TH-positive neurons with p-RIPK3 puncta was quantified. (*N* = 3 mice per genotype). **n** The proportion of TH-positive neurons with p-MLKL puncta was quantified. (*N* = 3 mice per genotype). Data were analyzed using two-way ANOVA followed by Sidak’s multiple comparisons tests (b, c, m, n) and Student’s t-test (e–g, i-k). Data are represented as the mean ± SEM. ^*^p < 0.05, ^***^p < 0.001, ^****^p < 0.0001

To further investigate the survival of DAergic neurons, we performed IFC staining for TH, a classic marker of DAergic neurons. We analyzed the number of DAergic neurons in the substantia nigra pars compacta (SNc) and ventral tegmental area (VTA) of *WDR45*^*cWT*^ and *WDR45*^*cKO*^ mice at young, middle-aged, and aged stages. We observed a reduction in the number of DAergic neurons in the VTA and SNc of middle-aged *WDR45*^*cKO*^ mice compared to age-matched *WDR45*^*cWT*^ mice, and this reduction was significantly exaggerated in the aged *WDR45*^*cKO*^ mice (Fig. 1a, b). Additionally, we found a significant decrease in the DA content in the SN of aged *WDR45*^*cKO*^ mice compared to that of aged *WDR45*^*cWT*^ mice (Fig. 1c).

We conducted further analysis to investigate the impact of WDR45-deficiency on DAergic neurons at the subcellular level. We used TEM analysis to assess mitochondrial morphology in aged *WDR45*^*cKO*^ mice. Our results showed the presence of vacuolized mitochondria in the neuronal soma from the SN region of aged *WDR45*^*cKO*^ mice but not in age-matched *WDR45*^*cWT*^ mice (Fig. 1d). The perimeter of mitochondria was significantly increased in *WDR45*^*cKO*^ mice compared to *WDR45*^*cWT*^ mice, and more than 40% of the mitochondria cristae of *WDR45*^*cKO*^ mice were broken or disappeared (Fig. 1e, f), indicating mitochondrial may damage upon WDR45-deficiency in DAergic neurons. The ER-mitochondria contact sites captured in the TEM images were analyzed, without significant alterations in the total number of their contact sites (Fig. S3a, b). The mitochondria in the striatum were also analyzed, and the proportion of mitochondria with damaged cristae (broken or completely vacuolized) was significantly increased (Fig. S3f, g). Moreover, the BCL2 Interacting Protein 3 (BNIP3, as a mitophagy receptor) positive puncta were upregulated in the striatum (Fig. S3c-e), indicating the accumulation of damaged mitochondria in the striatal region, where receives the projections from midbrain DAergic neurons. We also analyzed the rough endoplasmic reticulum (RER) structure and found a significant change in RER morphology in aged *WDR45*^*cKO*^ mice (Fig. 1h). The mean width of RER tubules was significantly expanded in the neurons from SN area of *WDR45*^*cKO*^ mice compared to that in *WDR45*^*cWT*^ mice (79.9 nm vs. 51.7 nm) (Fig. 1i). The proportion of RER tubules (> 100 nm) was increased from 6.4% in the aged *WDR45*^*cWT*^ mice to 23.9% in the *WDR45*^*cKO*^ mice (Fig. 1j), indicating that the DAergic neurons may suffer from severe RER tubular expansion due to *WDR45* deletion. The proportion of mitochondria with broken cristae or completely vacuolized were significantly increased (Fig. 1f). Moreover, the mean number of mitochondria captured in TEM images was also dramatically increased in the *WDR45*^*cKO*^ mice compared to that in the *WDR45*^*cWT*^ mice (Fig. 1g), which was further verified by the significant increase of the intensity of translocase of outer mitochondrial membrane 20 (TOM20) in the soma of DAergic neurons detected by IF analysis (Fig. S4a, b). All these findings indicated an impaired mitochondrial homeostasis, possibly due to the damaged mitochondria accumulation during the WDR45 deficiency-induced impaired autophagic process. Similarly, in addition to the increased number of swollen RER tubules (Fig. 1i, j), the mean number of captured RER tubules in TEM images was also remarkably increased (Fig. 1k). The presence of lysine-aspartic acid-glutamic acid-leucine (KDEL) is necessary for ER retention and to be sufficient to reduce the secretion of proteins from the ER. We also found that KDEL expression dramatically increased in the DAergic neurons of the *WDR45*^*cKO*^ mice (Fig. S4f). Additionally, to further clarify whether the associated functions of mitochondria and ER were impacted in the DAergic neurons, we detected several protein expressions in the DAergic neuronal soma by IFC staining and found BNIP3 expression in the nuclei was dramatically enhanced in the young *WDR45*^*cKO*^ mice compared to that in the young *WDR45*^*cWT*^ mice (Fig. S4b). However, in the aged mice, the BNIP3 positive puncta were mainly concentrated in the DAergic cytoplasm, with a significant increase in the *WDR45*^*cKO*^ mice (Fig. S4b). Additionally, the mitochondrial fission processes were predominantly impacted under the WDR45 deficit since the intensity of fission mitochondrial 1 (FIS1), the key protein that mediates the mitochondrial fission process, was markedly increased in the DAergic neurons of both young and aged *WDR45*^*cKO*^ mice (Fig. S4c-e). ER-associated proteins were also detected to examine the ER relative function, including SEC16 Homolog A, Endoplasmic Reticulum Export Factor (SEC16A, has a key role in the organization of the ER exit site), and SEC31 Homolog A, COPII Coat Complex Component (SEC31A, as the component of the coat protein complex II, which promotes the formation of transport vesicles from the ER). The expression of SEC16A in the DAergic neurons was significantly increased in the young *WDR45*^*cKO*^ mice, while SEC31A expression down-regulated dramatically in both young and aged *WDR45*^*cKO*^ mice, indicating the WDR45 deficit has an impact on the secretion and transport for proteins from ER (Fig. S4g, h). Overall, these findings suggest that *WDR45* deletion may inhibit the clearance or turnover of damaged organelles, accelerating the degeneration of DAergic neurons.

Given that the *WDR45*^*cKO*^ mice experience progressive reduction of DAergic neurons during aging, we aimed to investigate the mechanisms underlying cell reduction. Necroptosis is a regulated form of necrosis and is considered a new mode of cell death. When necroptosis is induced, receptor-interacting protein kinase-3 (RIPK3) becomes activated through phosphorylation and then phosphorylated RIPK3 activates mixed lineage kinase-like (MLKL) through phosphorylation [30, 31]. To determine if necroptosis was activated in the DAergic neurons of the *WDR45*^*cKO*^ mice, we assessed the presence of phosphor-RIPK3 and phosphor-MLKL puncta in neuronal cytoplasm using our previously established methods [32]. Our data revealed a marked increase in the concentrated puncta of phosphor-RIPK3 and phosphor-MLKL in the cytoplasm of middle-aged and aged *WDR45*^*cKO*^ mice (Fig. 1l, m, n). These results indicate that necroptosis was activated in the DAergic neurons, leading to progressive DAergic neuronal reduction in the *WDR45*^*cKO*^ mice during aging.

### Axonal degeneration in the striatum of WDR45^cKO^ mice

**Fig. 2:**
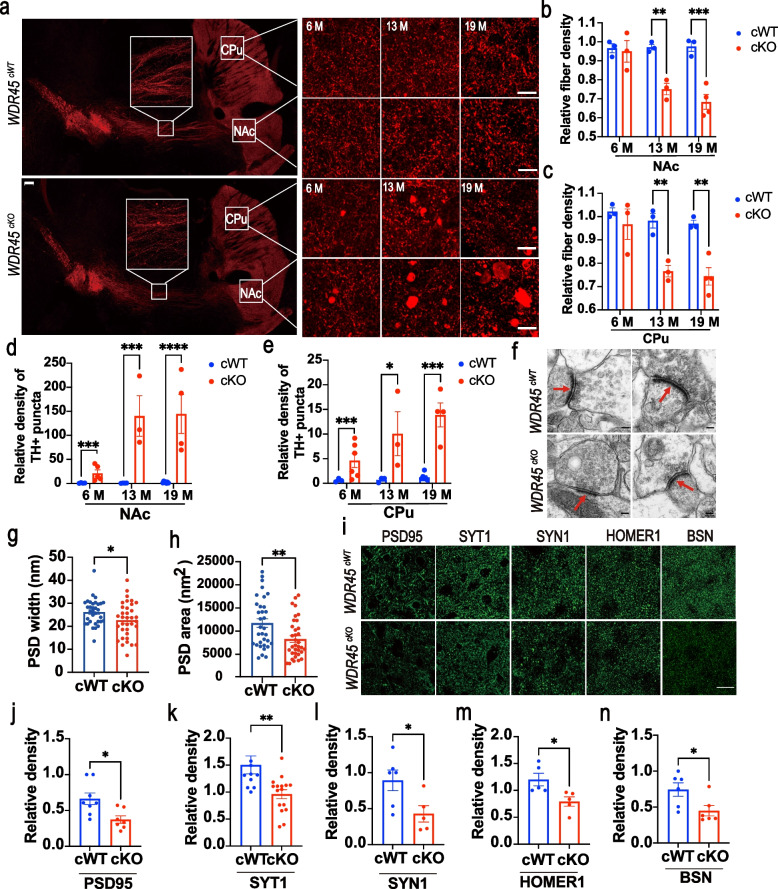
Axonal degeneration in the striatum of *WDR45*^*cKO*^ mice. **a** IFC staining for striatal axons, including the NAc and CPu, was performed using an antibody against TH (red) in young, middle-aged, and aged *WDR45*^*cWT*^ and *WDR45*^*cKO*^ mice. Scale bar, 200 μm. For high-magnification images: 50 μm. **b**, **c** Quantifying the fiber density in the NAc and CPu, respectively (*N* = 3 mice per genotype). **d**, **e** The calculation of densities of DA axonal enlargements (area > 5 μm^2^) per 0.045 mm^2^ perspective in the NAc and CPu from *WDR45*^*cWT*^ and *WDR45*^*cKO*^ mice, respectively (*N* = 3–7 slices from 3 mice per genotype). **f** Representative TEM images of the observed PSD. Scale bar, 100 nm. The red arrowhead indicates PSD. **g**, ** h** The PSD width and PSD area were quantified (*N* = 33 PSD collectively counted from 3 *WDR45*^*cWT*^ mice and 35 PSD from 3 *WDR45*^*cKO*^ mice). **i** IFC analysis of synapse-related proteins in the striatum of aged *WDR45*^*cWT*^ mice and *WDR45*^*cKO*^ mice. Scale bar, 20 μm. **j** Quantifying PSD95' fluorescence density (*N* = 7–8 slices from 3 mice per genotype). **k** Quantifying SYT1' fluorescence density (*N* = 11 slices from 3 mice per genotype). **l** Quantifying SYN1' fluorescence density (*N* = 5–6 slices from 3 mice per genotype). **m** Quantifying HOMER1' fluorescence density (*N* = 5 slices from 3 mice per genotype). **n** Quantifying BSN' fluorescence density (*N* = 7–8 slices from 3 mice per genotype). Data (b-e) were analyzed using two-way ANOVA followed by Sidak’s multiple comparisons test and Student’s t-test (g, h, j-n). Data are represented as the mean ± SEM. ^*^p < 0.05, ^**^p < 0.01, ^***^p < 0.001, ^****^p < 0.0001

In addition to the reduction of DAergic neurons in the SNc, *WDR45* deletion also led to profound nerve fiber pathology in the striatum. Our longitudinal study revealed substantial changes in DAergic axonal terminals projected to the striatum of *WDR45*^*cKO*^ mice. Specifically, we observed significant axonal enlargements in the young, middle-aged, and aged *WDR45*^*cKO*^ mice, along with reduced fiber density during aging (Fig. 2a-c). It is worth noting that significantly more enlargements were accumulated in the nucleus accumbens (NAc), which receives the projection of DAergic neurons from the VTA, than in the caudate putamen (CPu) from SNc DAergic neurons, in the young, middle-aged, and aged *WDR45*^*cKO*^ mice (Fig. 2d, e). These findings demonstrate that *WDR45*^*cKO*^ mice develop severe DAergic axonal degeneration in the striatum prior to neuronal loss and reveal the differential axonal vulnerability of DAergic neuronal subtypes in response to *WDR45* deletion.

Since large enlargements were observed in the axonal terminals, we decided to examine whether the striatal synapses were affected. To study the effect of *WDR45* deletion on excitatory synapses of DAergic projections, we conducted TEM analysis for postsynaptic density (PSD), which contributes to information processing and memory formation by changing synaptic strength in response to neural activity [33]. The results showed that PSD density was significantly reduced (Fig. 2f), and PSD width and average area were significantly reduced (Fig. 2g, h). The data suggest that the synaptic structures in the striatum of *WDR45*^*cKO*^ mice have undergone alterations. Furthermore, we assessed the levels of some synaptic proteins in the striatum, including PSD95, a membrane protein of presynaptic vesicles called synaptotagmin 1 (SYT1), a synaptic vesicle protein called synapsin-1 (SYN1), postsynaptic density scaffolding protein called homer scaffold protein 1 (HOMER1), and presynaptic cytomatrix protein bassoon (BSN). We observed a significant decrease in the expression of presynaptic-related proteins, BSN and SYT1, in the striatum of young *WDR45*^*cKO*^ mice (Fig. S5e), and a significant decrease in the fluorescence density of PSD95, SYT1, SYN1, HOMER1, and BSN in the striatum of aged ones (Fig. 2i-n), indicating the progressive dysfunction of synapses under WDR45 deficit in aging. Additionally, we detected the associated proteins of DAergic axonal terminals, including DAT, dopamine receptors D1 and D2 (DRD1, DRD2), and vesicular monoamine transporter member 2 (vMAT2). The expression of striatal DRD1 was significantly decreased, while DRD2 expression was dramatically increased in the aged *WDR45*^*cKO*^ mice (Fig. S5a-d), which may be associated with depression-like behavior. These results further support that synaptic signaling transmission is disrupted under the longtime dysfunction of WDR45.

### Accumulation of increased fragmented tubular ER constitutes a pathological feature of swollen axons in the WDR45^cKO^ mice

**Fig. 3:**
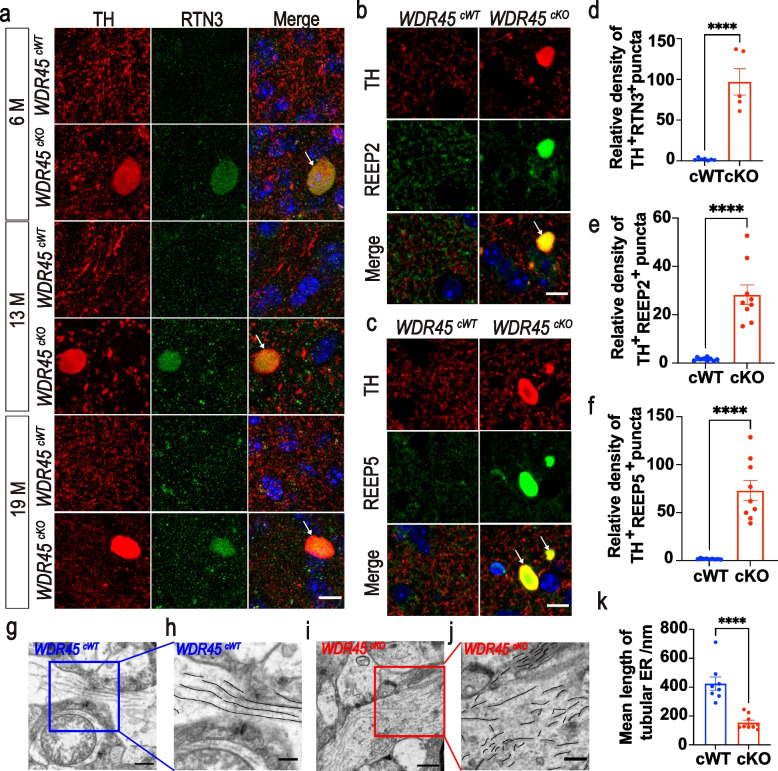
Increasing fragmented tubular ER constitutes a pathological feature of axons in *WDR45*^*cKO*^ mice. **a** IFC analysis for RTN3 in the NAc of *WDR45*^*cWT*^ mice and *WDR45*^*cKO*^ mice was performed using antibodies against RTN3 (green) and TH (red). The nuclei were labeled with DAPI (blue). Scale bar, 10 μm. **b** IFC staining for REEP2 in the NAc of aged *WDR45*^*cWT*^ mice and *WDR45*^*cKO*^ mice was performed using antibodies against REEP2 (green) and TH (red). The nuclei were labeled with DAPI (blue). Scale bar, 10 μm. **c** IFC staining for REEP5 in the NAc of aged *WDR45*^*cWT*^ mice and *WDR45*^*cKO*^ mice was performed using antibodies against REEP5 (green) and TH (red). The nuclei were labeled with DAPI (blue). Scale bar, 10 μm. **d** Analysis of relative density of RTN3- and TH-positive enlargements in the NAc of aged *WDR45*^*cWT*^ mice and *WDR45*^*cKO*^ mice (*N* = 5–9 slices from 3 mice per genotype). **e** Analysis of relative density of REEP2- and TH-positive enlargements in the NAc of aged *WDR45*^*cWT*^ mice and *WDR45*^*cKO*^ mice (*N* = 9 slices from 3 mice per genotype). **f** Analysis of relative density of REEP5- and TH-positive enlargements in the NAc of aged *WDR45*^*cWT*^ mice and *WDR45*^*cKO*^ mice (*N* = 9 slices from 3 mice per genotype). **g-j** Samples from aged *WDR45*^*cWT*^ mice and *WDR45*^*cKO*^ mice were examined by TEM, and representative TEM images of observed tubular ER at the axons of the striatum are shown. The tubular ER is highlighted in black. Scale bar, 500 nm. For enlarged images, 250 nm. **k** The mean length of tubular ER was analyzed from aged *WDR45*^*cWT*^ mice and *WDR45*^*cKO*^ mice (*N* = 8–9 slices from 3 mice for each genotype). Data were analyzed by using Student’s t-test. Data are represented as the mean ± SEM. ^****^p < 0.0001. White arrows indicate axonal enlargements

Axonal swellings (also called axonal beading, bubbling, or spheroid) are hallmarks of degenerating axons, almost universal in neurodegenerative diseases [34, 35]. In our study, *WDR45* depletion in the DAergic neurons resulted in axonal swellings in the striatum. To gain insights into the molecular basis of axonal degeneration, we evaluated potential candidates by investigating their presence at the axonal enlargements. First, we examined the ER proteins KDEL and cytoskeleton-associated protein 4 (CKAP4, also called Climp-63) [36], the tubular ER protein ATL3, and the tubular ER-shaping proteins RTN3 and RTN4 [37, 38]. RTN4, KDEL, Climp-63, and ATL3 were not observed in the axonal enlargements (Fig. S6). By contrast, RTN3 was highly concentrated at the striatal axonal enlargements in young, middle-aged, and aged *WDR45*^*cKO*^ mice (Fig. 3a, d), suggesting that RTN3 is one of the enlargement components and may contribute to the formation of axonal swellings as an early pathogenic event. As a typical tubular ER-shaping protein, the accumulation of RTN3 implies that the tubular ER shape may be affected in the striatum. We then investigated the molecular composition of axonal enlargements by determining other tubular ER-shaping proteins, REEP2 and REEP5 [39, 40]. We found that REEP2 and REEP5 also colocalized with TH-positive enlargements (Fig. 3b, c, e, f), further indicating that the shape of tubular ER in the axons was disrupted upon *WDR45* depletion. These findings highlight the crucial role of ER-shaping proteins in forming axonal enlargements, providing further evidence for the importance of maintaining a normal tubular ER shape in regulating distal axonal homeostasis. The above findings prompted us to determine whether the tubular ER shape is abnormal in the *WDR45*^*cKO*^ mice. We then examined the tubular ER ultrastructure by TEM in the striatal samples from aged *WDR45*^*cWT*^ and *WDR45*^*cKO*^ mice. Compared to the normally distributed tubular ER in *WDR45*^*cWT*^ mice, a remarkably large accumulation of fragmented tubular ER was noticed in the axons of *WDR45*^*cKO*^ mice (Fig. 3g-k), supporting the notion that the fragmented tubular ER cluster is a major pathological abnormality associated with axonal degeneration in *WDR45*^*cKO*^ mice.

### Disrupted autophagic flux in the DAergic neurons may contribute to the accumulation of tubular ER in axons

**Fig. 4:**
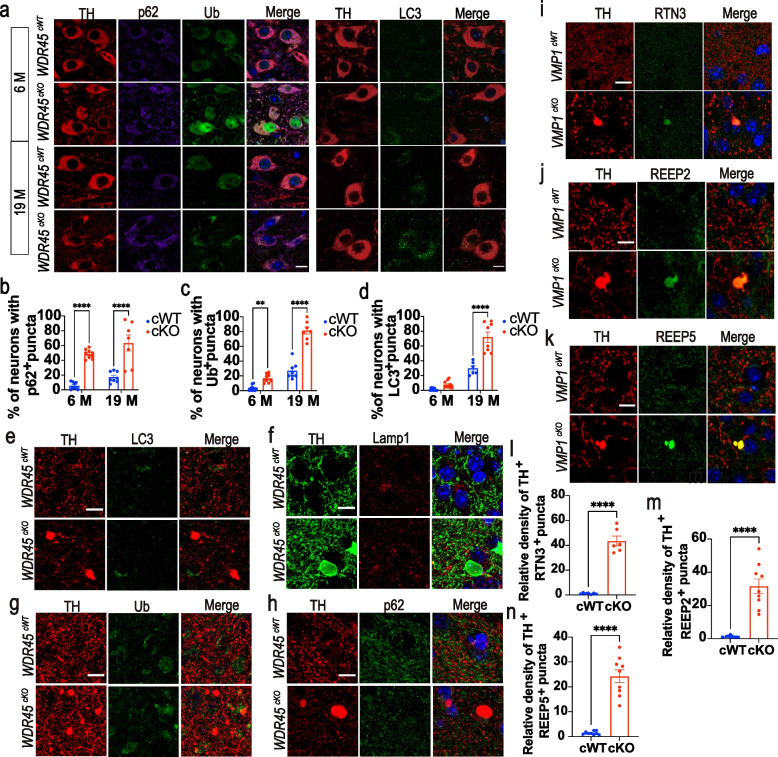
Disrupted autophagic flux in the DAergic neurons may contribute to the accumulation of tubular ER in axons. **a** Left panel: IFC staining for p62 (purple) and Ub (green) in the TH-positive neurons (red) of *WDR45*^*cWT*^ mice and *WDR45*^*cKO*^ mice. The nuclei were labeled with DAPI (blue). Scale bar, 10 μm. Right panel: IFC staining for LC3 (green) in the TH-positive (red) DAergic neurons. The nuclei were labeled with DAPI (blue). Scale bar, 10 μm. **b** The proportion of TH-positive neurons with p62 puncta (> 0.5 μm^2^) is presented (*N* = 3 mice per genotype). **c** The proportion of TH-positive neurons with Ub-positive puncta (> 0.5 μm^2^) is presented (*N* = 3 mice per genotype). **d** The proportion of TH-positive neurons with LC3-positive puncta (> 0.5 μm^2^) is presented (*N* = 3 mice per genotype). **e–h** LC3 (green), Lamp1 (red), Ub (green), and p62 (green) were detected in the NAc of aged *WDR45*^*cWT*^ mice and *WDR45*^*cKO*^ mice. The nuclei were labeled with DAPI (blue). Scale bar, 10 μm. **i-k** IFC staining of RTN3, REEP2, and REEP5 in the NAc of 12-month-old *VMP1*^*cWT*^ mice and *VMP1*^*cKO*^ mice was performed using antibodies against RTN3 or REEP2 or REEP5 (green) with TH (red), respectively. The nuclei were labeled with DAPI (blue). Scale bar, 10 μm. **l-n** Analysis of the relative density of RTN3- and TH-positive enlargements, REEP2- and TH-positive enlargements, and REEP5- and TH-positive enlargements (> 5 μm^2^), respectively (*N* = 5–9 slices from 3 mice per genotype). Data were analyzed using two-way ANOVA followed by Sidak’s multiple comparisons tests (b-d) and Student’s t-test (l-n). Data are represented as the mean ± SEM. ^****^p < 0.0001, ^**^p < 0.01

To understand what contributes to the accumulation of tubular ER at axons, we first examined whether autophagic flux was disrupted in the DAergic neurons of *WDR45*^*cKO*^ mice. We stained midbrain sections to detect the expression and distribution of autophagic substrates SQSTM1 (p62) and Ub, as well as LC3 (a classical marker for autophagic vesicles). Compared with *WDR45*^*cWT*^ mice, we observed distinct p62-positive puncta accumulated in the soma of DAergic neurons in young *WDR45*^*cKO*^ mice, and this accumulation was aggravated in the aged *WDR45*^*cKO*^ mice (Fig. 4a, b). Additionally, we found that Ub expression was significantly increased in the nucleus of DAergic neurons of young *WDR45*^*cKO*^ mice and in the cytoplasm of DAergic neurons of aged *WDR45*^*cKO*^ mice, of which the Ub staining was not entirely colocalized with p62-positive puncta (Fig. 4a, c). Similarly, LC3-positive puncta were also concentrated in the cell body of DAergic neurons in the aged *WDR45*^*cKO*^ mice (Fig. 4a, d). These data suggest that *WDR45* depletion induced an early impairment of autophagic flux in the DAergic neurons, likely triggering axonal and cell body degeneration.

To assess whether LC3-labeled autophagosomes are present in axonal enlargements, we stained the striatal sections and found that LC3-positive puncta were absent in the TH-positive axonal enlargements (Fig. 4e), indicating that the LC3-labeled autophagosomes did not directly contribute to the formation of axonal enlargements. Furthermore, the lysosome marker Lamp1 was also absent in the axonal enlargements (Fig. 4f). The autophagic substrates Ub and p62 were also not colocalized with axonal enlargements (Fig. 4g, h). Therefore, the accumulations of autophagic proteins were mainly observed in the soma but not in the axons of DAergic neurons deficient in WDR45. We speculate that disrupting autophagic flux in the DAergic neurons may lead to axonal enlargements by promoting tubular ER accumulation at axons. To test this hypothesis, we employed another mouse model with damaged autophagic flux, the *VMP1*^*cKO*^ mice that conditionally knocked out autophagic gene *VMP1* in the DAergic neurons upon TAM treatment postnatally [32]. *VMP1*^*cKO*^ mice also displayed severe damage to autophagic flux and large axonal enlargements in the striatum [32]. RTN3, REEP2, and REEP5 were highly accumulated at the TH-positive axonal enlargements in the striatum of 12-month-old *VMP1*^*cKO*^ mice (Fig. 4i-n), indicating that defective autophagy may induce axonal accumulation of tubular ER. Together, these results suggest that the abnormal clustering of tubular ER in axons may have pathological effects on the brain. Furthermore, our findings provide additional evidence that autophagy plays a critical role in maintaining axonal homeostasis by regulating the shape and accumulation of tubular ER.

### Exploring the proteome landscape *of striatum* in the *WDR45 *^*cKO*^ mice

**Fig. 5:**
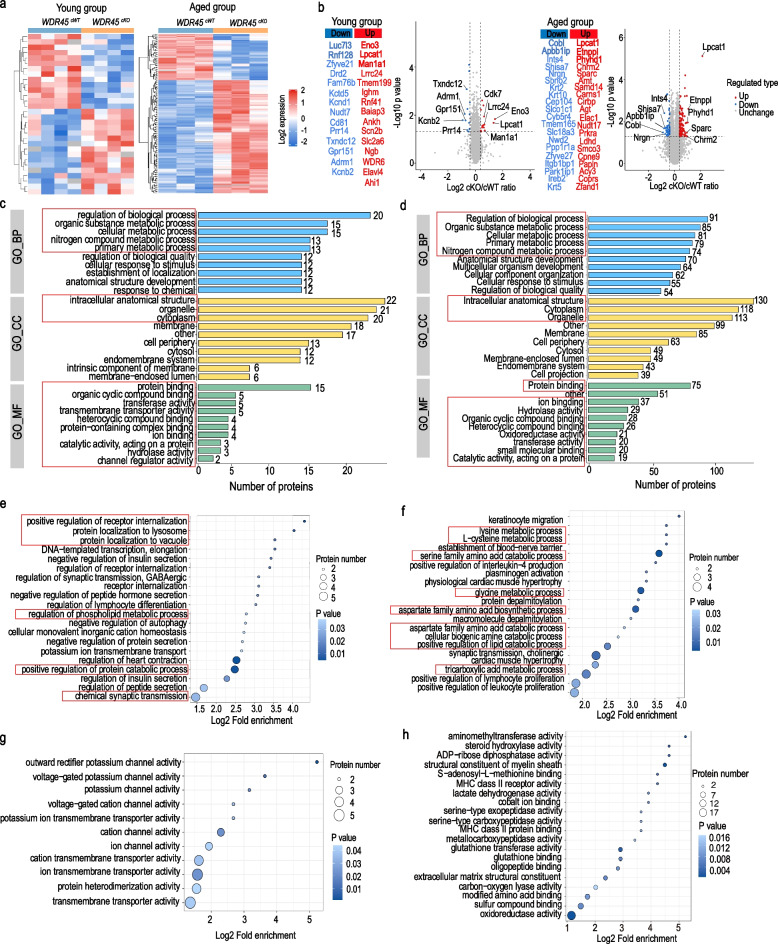
The proteome landscape of striatum in the *WDR45 *^*cKO*^ mice. **a** The heatmap of the DEPs from young and aged *WDR45*^*cWT*^ mice and *WDR45*^*cKO*^ mice. **b** Volcano plots and top 20 up- or down-regulated DEPs organized by fold change in the striatum of young and aged *WDR45*^*cKO*^ mice vs. *WDR45*^*cWT*^ mice (DEPs marked by red and blue circles). The top 10 CC, MF, and BP terms in GO annotation analysis for DEPs from (**c**) young and (**d**) aged *WDR45*^*cWT*^ mice and *WDR45*^*cKO*^ mice. The top 20 terms related to BP in the GO enrichment analysis for DEPs from (**e**) young and (**f**) aged *WDR45*^*cWT*^ mice and *WDR45*^*cKO*^ mice. The top 20 terms related to MF in the GO enrichment analysis for DEPs from (**g**) young and (**h**) aged *WDR45*^*cWT*^ mice and *WDR45*^*cKO*^ mice. Data were analyzed by using the Student’s t-test. Data are represented as the mean ± SEM. ^****^p < 0.0001

To gain a general landscape of the pathological abnormalities in the DAergic axons, we dissected the striatal samples from both young and aged *WDR45*^*cKO*^ mice, age- and sex-matched *WDR45*^*cWT*^ mice for proteomic analysis. Principal component analysis (PCA) revealed the distinct proteomic profiles of *WDR45*^*cWT*^ mice and *WDR45*^*cKO*^ mice (Fig. S7a, b). Further proteomic analyses registered 6,647 targets in the young mice and 6,290 targets in the aged mice, of which 31 differentially expressed proteins (DEPs) in the young mice and 167 DEPs in the aged mice were identified (> 1.3-fold or < 1/1.3-fold change cutoff, *p* < 0.05). Among the DEPs, 17 from young mice were upregulated, 14 were downregulated, 115 DEPs from aged mice were upregulated, and 47 were downregulated (Fig. S7c, d, and Supplementary Table 1). In the young mice, the majority of the top 10 up-regulated proteins are involved in the regulation of carbohydrate metabolic process, such as Beta-enolase3 (Eno3), and in the regulation of lipid metabolic process, like lysophosphatidylcholine acyltransferase 1 (Lpcat1), as well as regulating nitrogen compounds' metabolic process, like mannosyl-oligosaccharide 1,2-alpha-mannosidase IA (Man1a1) (Fig. 5a, b). The top 10 downregulated DEPs are most associated with nucleobase-containing compound metabolic process, regulation of cytokine production, and nitrogen compounds' metabolic process (Fig. 5a, b). In the aged mice, the majority of the top 20 up-regulated proteins were enzymes that are involved in the regulation of lipid metabolic process, such as Lpcat1, ethanolamine-phosphate phospho-lyase (Etnppl), abhydrolase domain containing 4, N-acyl phospholipase B (Abhd4), and phytanoyl-CoA dioxygenase domain-containing protein 1 (Phyhd1), and in the regulation of nitrogen compounds' metabolic process, such as ElaC ribonuclease Z1 (Elac1), aminomethyltransferase (Amt), lactate dehydrogenase D (Ldhd), and cold-inducible RNA binding protein (Cirbp), as well as in regulating anatomical structure morphogenesis, like secreted protein acidic and cysteine-rich (Sparc), angiotensinogen (Agt) (Fig. 5a, b). The top 20 downregulated DEPs are most associated with nitrogen compounds' metabolic process, such as complex integrator subunit 4 (Ints4), keratin 2 (Krt2), and strawberry notch homolog 2 (Sbno2), and proteins with cell morphogenesis, such as protein cordon-bleu (Cobl), amyloid β-A4 precursor protein-binding family B member 1-interacting protein (Apbb1ip) (Fig. 5a, b).

To support the biological significance of these DEPs, we performed Gene Ontology (GO) annotation analysis, which depicts protein functions in three categories: biological processes (BP), cellular components (CC), and molecular functions (MF). The most correlated BP of the DEPs from both young and aged mice is the regulation of the biological process, metabolic-related process, including organic substance, cellular, primary, and nitrogen compounds metabolic process, and that regulation of anatomical structure development (Fig. 5c, d). Regarding CC, these DEPs are mostly found in the intracellular anatomical structure, cytoplasm, and organelle (Fig. 5c, d). The analysis results of MF show that these DEPs are primarily associated with protein binding, ion binding, organic cyclic compound binding, and hydrolase activity, etc. (Fig. 5c, d). To further investigate the functions and signaling pathways of the DEPs, we performed GO enrichment analyses. In the young mice, the top 20 enriched BP pathways are most related to positive regulation of receptor internalization, protein localization to lysosome and vacuole, regulation of phospholipid metabolic process, and positive regulation of protein catabolic process (Fig. 5e). While in the aged group, amino acids' catabolic and biosynthetic processes, positive regulation of the lipid catabolic process, and protein depalmitoylation are the most enriched pathways (Fig. 5f). Notably, many DEPs from young mice are involved in regulating ion channel activity and transmembrane transporter activity, like outward rectifier potassium channel activity and voltage-gated potassium channel activity, cation channel activity, and ion transmembrane transporter activity (Fig. 5g). As aging progresses, the regulation of DEPs is mostly involved in the enzyme activity, like oxidoreductase activity, Amt, and steroid hydroxylase, that are enriched in the amino acid metabolic process pathway, including lysine, L-cysteine, serine family amino acids, glycine, aspartate family amino acids, and in the tricarboxylic acid metabolic process, lipid catabolic process (Fig. 5h).

Overall, the proteomic data indicate that after WDR45 deficiency in the DAergic neurons, proteins that regulate ion transmembrane transporter activity and ion channel activity change earlier. The main changes brought by these proteins focus on regulating receptor internalization, protein localization and catabolism, and chemical synaptic transmission. The impact of WDR45 deficiency becomes more profound with aging. The proteins that undergo changes mainly focus on those that regulate enzyme activity, such as oxidoreductase activity, Amt activity, and glutathione transferase activity, mainly affecting amino acid and lipid metabolism, and there may be severe oxidative stress reactions in the brain. The activated catabolism of these molecules may also indicate an energy supply deficit in the striatum, supported by the enrichment of DEPs in the tricarboxylic acid metabolic process that produces adenosine triphosphate (ATP) for cellular energy (Fig. 5h).

### The connection of the phospholipid metabolism with the striatal pathology

**Fig. 6:**
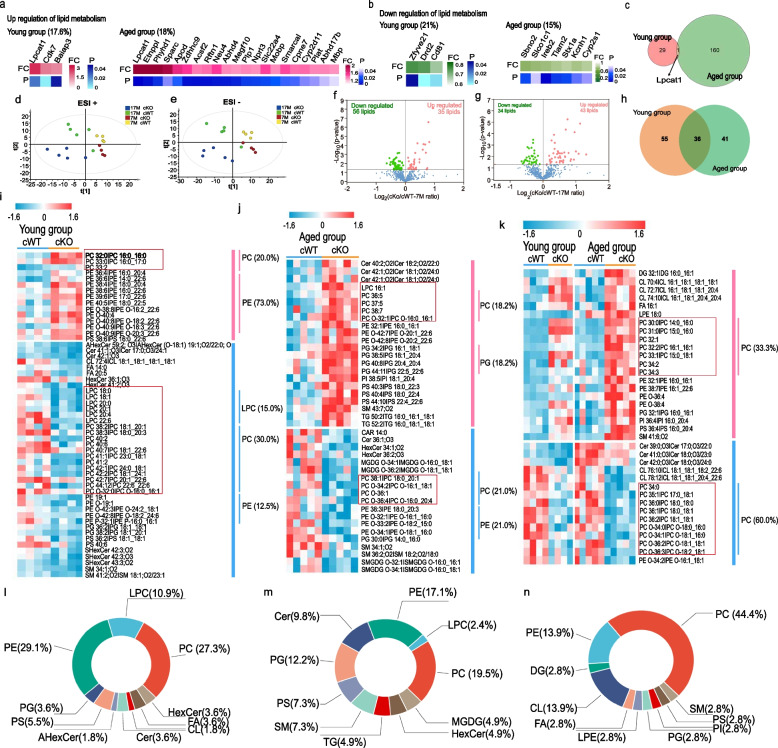
The connection of the phospholipid metabolism with the striatal pathology. GO analysis for BP displays up-regulated DEPs (pink) (**a**) and downregulated DEPs (green) (**b**) that participate in the regulation of lipid metabolism. Lower blue bars represent the magnitude of *p* values. Percentages indicate the fraction of each category of total up- or down-regulated DEPs. **c** The Venn diagram of the number of DEPs in young and aged groups. **d** PLS-DA score plot of the lipidomic profile of striatum tissues from both young and aged *WDR45*^*cKO*^ mice and *WDR45*^*cWT*^ mice in positive mode. R2X = 0.454, R2Y = 0.492, Q2 = 0.219. **e** PLS-DA score plot of the lipidomic profile of striatum tissues from both young and aged *WDR45*^*cKO*^ mice and *WDR45*^*cWT*^ mice in negative mode. R2X = 0.518, R2Y = 0.510, Q2 = 0.271. **f** Volcano plot of DELs between young *WDR45*^*cKO*^ mice and *WDR45*^*cWT*^ mice. **g** Volcano plot of DELs between aged *WDR45*^*cKO*^ mice and *WDR45*^*cWT*^ mice. **h** Venn diagram of the number of DELs between *WDR45*^*cKO*^ mice and *WDR45*^*cWT*^ mice in young and aged groups. Heat maps of DELs between *WDR45*^*cKO*^ mice and *WDR45*^*cWT*^ mice in (**i**) young groups, (**j**) aged group, and (**k**) both young and aged groups. Percentage of DELs in each lipid class between *WDR45*^*cKO*^ mice and *WDR45*^*cWT*^ mice in (**l**) young groups, (**m**) aged group, and (**n**) both young and aged groups

According to the proteomic data, 17.6% of up-regulated DEPs in young mice and 18% in aged mice were found to regulate lipid metabolism. Additionally, among down-regulated DEPs, 21% from young mice and 15% from aged mice were involved in lipid metabolism (Fig. 6a, b). To validate these findings from proteomics, we performed qRT-PCR and confirmed the role of lipid metabolism in striatal pathology (Fig. S7e, f). Notably, the expression of Lpcat1 (also called AYTL2), a phospholipid biosynthesis/remodeling enzyme that facilitates the conversion of palmitoyl-lysophosphatidylcholine (PPC) to dipalmitoyl-phosphatidylcholine (1,2-dipalmitoyl-sn-glycero-3-phosphocholine, DPPC), was significantly increased both in young and aged *WDR45*^*cKO*^ mice (Fig. 6c). To gain further insights into the association between striatal pathology and lipids, particularly phospholipids, we performed a comprehensive lipidomic analysis with a specific focus on phospholipid metabolites. The striatal lipid profile of both young and aged *WDR45*^*cKO*^ mice exhibited distinct separation from that of *WDR45*^*cWT*^ mice in both positive and negative ionization modes, indicating a significant alteration in the striatal lipid profile due to the loss of *WDR45* in DAergic neurons (Fig. 6d, e).

Further lipidomic analyses identified 91 differentially expressed lipids (DELs) in young mice and 77 DELs in aged mice. Among the DELs from young *WDR45*^*cKO*^ mice, 56 were down-regulated and 35 were up-regulated compared to young *WDR45*^*cWT*^ mice, while in aged *WDR45*^*cKO*^ mice, 34 DELs were down-regulated and 43 were up-regulated compared to aged *WDR45*^*cWT*^ mice (Fig. 6f, g, and Supplementary Table 2). Additionally, there were 36 DELs shared by both age groups (Fig. 6h). In detail, we identified a total of 12 categories of DELs in young mice including phosphatidylcholine (PC), phosphatidylethanolamine (PE), lysophosphatidylcholine (LPC), phosphatidylglycerol (PG), phosphatidylserine (PS), acylhexosylceramide (AHexCer), ceramide (Cer), cardiolipin (CL), fatty acid (FA), sphingomyelin (SM), hexosyl ceramide (HexCer) and its sulfatide (SHexCer) (Fig. 6i). According to the phosphorylated substituents at the C-3 position of glycerol, glycerophospholipids can be divided into PC, PE, PS, PG, LPC, etc. PE, as well as other glycerophospholipids, is diverse according to the different combinations of fatty acids of varying lengths and saturation attached at the C-1 and C-2 positions. In this study, we provided detailed DELs. Among the 15 up-regulated DELs, there are mainly 11 PEs and 3 PCs, accounting for 73% and 20% respectively (Fig. 6i). Of note, the catalytic product of Lpcat1 PC 32:0|PC 16:0_16:0 is significantly increased in the young *WDR45*^*cKO*^ mice. 40 DELs were downregulated, mainly including 6 LPCs, 12 PCs, and 5 PEs, with a proportion of 15%, 30%, and 12.5%, respectively (Fig. 6i). In aged mice, a total of 13 categories of DELs were identified, including PC, LPC, PE, Cer, PG, PI, PS, SM, TG, acylcarnitine (CAR), HexCer, monogalactosyl diglyceride (MGDG), etc. (Fig. 6j). Compared to young mice which had 12 categories of DELs, the aged mice exhibited a total of 13 categories of DELs. The proportion within each category also showed alterations. Among the up-regulated DELs in aged mice, PCs and PGs accounted for the highest proportion at 18.2% each, whereas among down-regulated DEL, PCs (21.0%) and PEs (21.0%) accounted for the highest proportion (Fig. 6j). Additionally, among the shared 36 DELs between young and aged mice, PCs were found to have the highest proportion accounting for 33.3% and 60% in up-regulated and down-regulated DELs, respectively (Fig. 6k). Overall, PCs emerged as predominant among these age-specific or common DELs with proportions of 27.3%, 19.5%, and 44.4% for young mice, aged mice, and shared DELs specifically (Fig. 6l-n).

### Disturbance of Lpcat1 expression ameliorates DAergic axonal degeneration

**Fig. 7:**
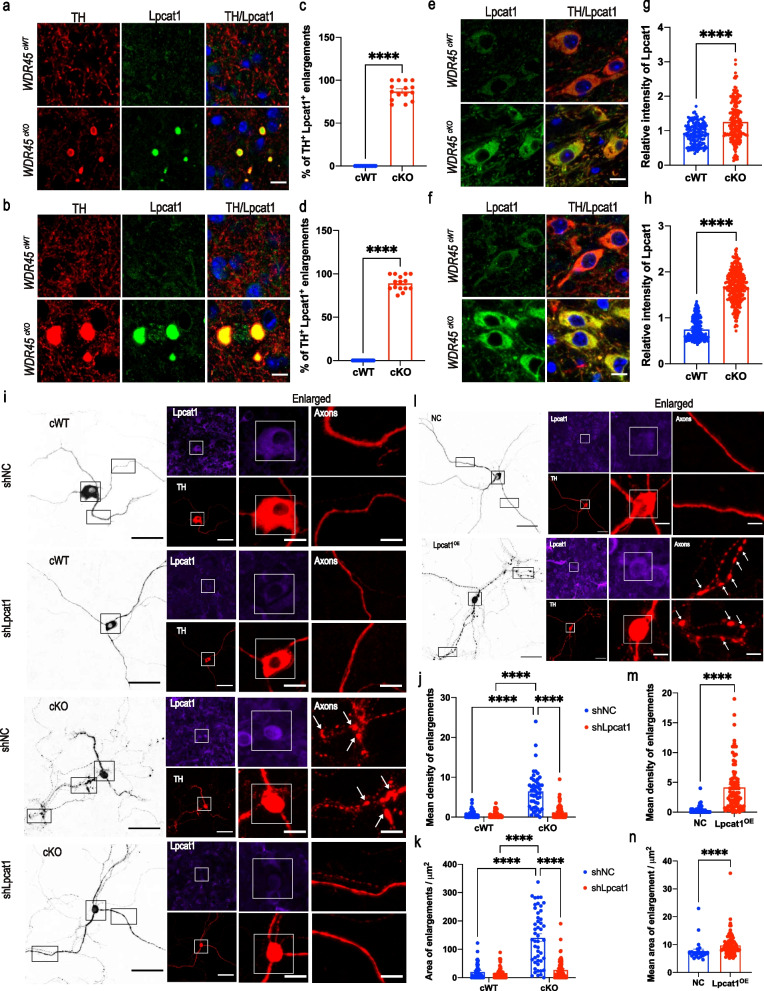
Disturbance of Lpcat1 expression ameliorates DAergic axonal degeneration. IFC staining for Lpcat1 in the striatum of young (**a**) and aged (**b**) *WDR45*^*cWT*^ mice and *WDR45*^*cKO*^ mice was performed using antibodies against Lpcat1 (green) and TH (red), and the analysis of Lpcat1- and TH-positive enlargements (**c**, **d**) (*N* = 15 slices from 3 mice per genotype). The nuclei were labeled with DAPI (blue). Scale bar, 10 μm. IFC analysis for Lpcat1 in the DAergic  in the SNc of young (**e**) and aged (**f**) *WDR45*^*cWT*^ mice and *WDR45*^*cKO*^ mice was performed using antibodies against Lpcat1 (green) and TH (red), and the analysis of relative fluorescence intensity for Lpcat1(**g, h**) (*N* = 141 neurons from 3 young *WDR45*^*cWT*^ mice, 183 neurons from 3 young *WDR45*^*cKO*^ mice, 198 neurons from 3 aged *WDR45*^*cWT*^ mice, and 281 neurons from 3 aged *WDR45*^*cKO*^ mice). The nuclei were labeled with DAPI (blue). Scale bar, 10 μm. **i** IFC staining for primary culture of DAergic neurons by using antibodies against Lpcat1 (pseudo-color) and TH (red), and the analysis of the mean density and total areas of enlargements per DAergic neuron (**j, k**) (*N* = 50–73 primary DAergic neurons collected from 3 P0 pups per group). Scale bar, 50 μm. Scale bar for high-magnification images, 20 μm. **l** IFC staining for primary culture of DAergic neurons by using antibodies against Lpcat1 (pseudo-color) and TH (red), and the analysis of the mean density and mean area of enlargements in each DAergic neuron (**m, n**) (*N* = 64–88 primary DAergic neurons collected from 3 P0 pups per group). Scale bar, 50 μm. Scale bar for high-magnification images, 20 μm. Data (j, k) were analyzed using two-way ANOVA followed by Tukey's multiple comparisons test, and Student’s t-test for data (c, d, g, h, m, n). Data are represented as the mean ± SEM. ^****^p < 0.0001. White arrows indicate axonal enlargements

Lpcat1 facilitates the conversion of PPC to DPPC [41]. Our lipidomic data showed that the PC 32:0IPC 16:0_16:0, also known as DPPC, was significantly upregulated in the striatum of *WDR45*^*cKO*^ mice (Fig. 6i), which was consistent with the dramatically increased Lpcat1 expression in the *WDR45*^*cKO*^ mice as shown by proteomic data. To further study the role of Lpcat1 in axonal pathology, we examined the Lpcat1 expression by performing WB and IF staining and found that Lpcat1 was distinctly increased in the striatum (Fig. S7g-i), and thoroughly concentrated in the DAergic axonal enlargements both in the young and aged *WDR45*^*cKO*^ mice (Fig. 7a-d), proving that the raised Lpcat1 contents mainly accumulated at the axonal enlargement and may play important roles in the axonal degeneration. To further investigate whether the increase of Lpcat1 expression is limited within the local axons or extends to the entire neuron, we detected the Lpcat1 expression in the midbrain where DAergic neurons soma reside. Unexpectedly, the expression of Lpcat1 was dramatically increased in the midbrain tissue homogenate (Fig. S7g-i) and raised significantly in the soma of DAergic neurons of both young and aged *WDR45*^*cKO*^ mice (Fig. 7e-h), indicating the increase of Lpcat1 is global in the neuron. These results show that Lpcat1 may be the key molecule in neuronal pathology, especially axonal degeneration induced by WDR45 deficiency. To further study whether Lpcat1 participates in the WDR45 deficiency-induced axonal degeneration, we conducted primary midbrain neuron cultures to detect the DAergic axonal pathology when the Lpcat1 expression was interfered by lentivirus transfection. At the DIV7, the LV-shLpcat1 or LV-shNC was added to transfect neurons for 24 h and replaced the culture medium at DIV8 with a collected cultured medium containing 1 μM 4-OHT to induce Cre recombinase activity. The neurons were collected at DIV13 for subsequent detection. Lpcat1 was efficiently downregulated after transfection for 5 days (Fig. S7j, k) and WDR45 was significantly decreased in the DAergic neurons following the treatment with 4-OHT (Fig. S7l, m). IF staining was performed and TH was co-stained to label DAergic neurons. We analyzed the mean densities of axonal enlargements in each DAergic neuron, as well as the total areas of axonal swellings per DAergic neuron. DAergic axons exhibit swellings under 4-OHT induced WDR45 deficiency, which mimics that in vivo (Fig. 7i). The axonal swellings in *WDR45*^*cKO*^ DAergic neurons presented a significant down-regulation in the size and quantity when inhibited Lpcat1 expression (Fig. 7i-k), indicating that Lpcat1 participates in the regulation of axonal degeneration induced by WDR45 defects. It seems that hyper-expression of Lpcat1 may bring harmful effects to axons. To verify this hypothesis, we used lentivirus to overexpress Lpcat1 in wildtype primary midbrain DAergic neuron cultures at DIV7 and at DIV11 the Lpcat1 level in DAergic neurons was examined by IF staining and WB. Lpcat1 was efficiently upregulated 4 days after transfection (Fig. S7n, o). We found that abundant, larger swellings appear in the DAergic axons in the Lpcat1-overexpressed (Lpcat1^OE^) group compared to that in the control (NC) group (Fig. 7l-n). These data strongly prove that Lpcat1 is the key regulator of axonal degeneration and provide an important clue for studying axonal degeneration in BPAN and other neurodegenerative diseases.

## Discussion

**Fig. 8:**
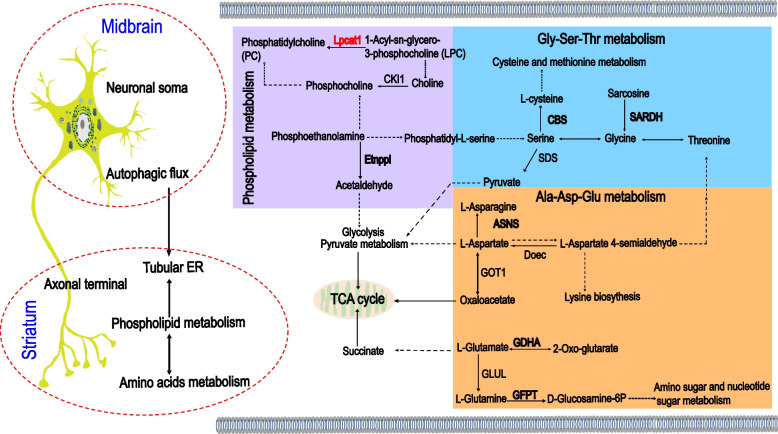
The potential interactions of autophagy, phospholipid metabolism, and tubular ER in the striatal pathology of WDR45-deficiency-induced DAergic neurodegeneration. The dysfunction of WDR45 impairs the DAergic neuronal autophagic process, an intracellular degradation system and compensation mechanism for energy supply, leading to protein accumulation and inhibition of damaged organelles turnover, then developing into the long-term metabolic disorders in striatal region and ultimately accelerating the axonal degeneration. The phospholipid metabolism interacts with amino acid metabolism and potentially regulates energy production. Additionally, the phospholipid metabolic process plays a role in the composition of tubular ER structural phospholipids. These complex interactions jointly regulate axonal homeostasis. The black bold texts represent the up-regulated DEPs from the current proteome analysis, and the red bold texts represent the DEPs we further examined experimentally. Black arrows show single-step enzyme catalysis. The black dashed arrows indicate multi-step enzyme catalysis. The right panel shows the perturbed pathways in phospholipid metabolism, Gly-Ser-Thr metabolism, Ala-Asp-Glu metabolism, and pyruvate metabolism, as well as the interactions among them. Abbreviations: Gly, glycine; Ser, serine; Thr, threonine; Ala, alanine; Asp, aspartate; Glu, glutamate; Lpcat1, lysophosphatidylcholine acyltransferase 1; CKI1, choline kinase1; Etnppl, ethanolamine-phosphate phospho-lyase; CBS, cystathionine beta-synthase; SDS, L-serine/L-threonine ammonia-lyase; SARDH, sarcosine dehydrogenase; ASNS, asparagine synthase; Doec, aspartate-semialdehyde dehydrogenase; GOT1, aspartate aminotransferase1; GDHA, glutamate dehydrogenase; GFPT, glutamine-fructose-6-phosphate transaminase; GLUL, glutamine synthetase

De novo heterozygous mutations in *WDR45* have been reported to cause BPAN, a subtype of neurodegeneration with brain iron accumulation, especially in the SN [42]. In the current study, we generated and characterized a mouse model *WDR45*^*cKO*^ which exhibited neurodegeneration of midbrain DAergic neurons, particularly axonal degeneration. We performed proteomic and lipidomic analysis to unlock proteome and lipidome profiles of the striatum, where the DAergic axonal terminals were projected from the midbrain. This profile represents the most comprehensive local omics profile associated with WDR45 deficiency-induced axonal degeneration published so far. Moreover, this study unveiled the interconnection between phospholipid metabolism, especially PC, with striatal pathology associated with WDR45 dysfunction in the DAergic system, provided a key molecule, Lpcat1, for the mechanism study of axonal degeneration in BPAN and other neurodegenerative diseases (Fig. 8).

The reduction of midbrain DAergic neurons in the *WDR45*^*cKO*^ mice is similar to the progressive DAergic neurodegeneration in BPAN patients [9]. In the current *WDR45*^*cKO*^ mice, the DAergic neuronal reduction was first observed in the middle-aged mice. Necroptosis marker p-MLKL shares a common expression pattern with BNIP3 and Ub. The increased p-MLKL in DAergic neurons was first observed in the nucleus before neuronal reduction when *WDR45*^*cKO*^ mice were at 6 months of age. Then, this p-MLKL was distributed to the cytosol of middle-aged and aged *WDR45*^*cKO*^ mice (Fig. 1l). The increase of cytoplasmic p-MLKL was parallel with neuronal death. A similar pattern was also observed in the BNIP3 and Ub expressions. The translocation of BNIP3 from the nucleus to cytosol indicates the initiation of the mitochondrial pathway of cellular death [43, 44]. This commonly shared pattern of these proteins’ expression may imply which stage the cell is undergoing. Such a phenomenon further supports that the DAergic neuronal stress in the *WDR45*^*cKO*^ mice is progressive and ultimately develops into cell death.

Axonal enlargement emerges as an early pathological event in axonal degeneration in Alzheimer's disease, Parkinson's disease, amyotrophic lateral sclerosis, and other neurodegenerative diseases [45–48]. However, the exact molecular mechanisms underlying are still poorly understood. Striatal axonal pathology occurs long before midbrain DAergic neuronal reduction in *WDR45*^*cKO*^ mice. The axonal degeneration in young *WDR45*^*cKO*^ mice initially manifested as axonal enlargement. Overall, striatal synaptic function became weak in *WDR45*^*cKO*^ mice, evidenced by the reduction of PSD95, SYT1, SYN1, HOMER1, and BSN (Table 3). Such a functional decline may result from DAergic axonal degeneration, long-term DA reduction, and insufficient postsynaptic action potential [45, 49].

**Table 3:** The summary for specific alterations of detected proteins in the current study

Processes	Detected proteins	Detected regions	Alterations in Expression (cKO/cWT)		Colocalized with TH^+^ axonal swellings?
			Young	Aged	
Neuronal degeneration	TH	SN	NS	Down	ND
	pRIPK3	SN	NS	Up	ND
	pMLKL	SN	NS	Up	ND
Axonal degeneration	TH	Striatum	NS	Down	Yes
Autophagic flux	P62	SN	Up	Up	ND
		Striatum	NS	NS	No
	Ub	SN	Up	Up	ND
		Striatum	NS	NS	No
	LC3	SN	NS	Up	ND
		Striatum	NS	NS	No
	Lamp1	Striatum	NS	NS	No
Synaptic functions	PSD95	Striatum	NS	Down	No
	SYT1	Striatum	Down	Down	No
	SYN1	Striatum	NS	Down	No
	HOMER1	Striatum	NS	Down	No
	BSN	Striatum	Down	Down	No
	DRD1	Striatum	NS	Down	No
	DRD2	Striatum	NS	Up	No
	DAT	Striatum	NS	NS	No
	vMAT2	Striatum	NS	NS	No
Mitochondrial functions	TOM20	SN	Up	Up	ND
	FIS1	SN	Up	Up	ND
	MFN1	SN	Up	NS	ND
	OPA1	SN	Up	NS	ND
	BNIP3	SN	Up	Up	ND
		Striatum	NS	NS	No
	NMNAT3	SN	NS	NS	ND
		Striatum	NS	NS	No
RER functions	SEC16A	SN	Up	NS	ND
	SEC31A	SN	Down	Down	ND
	KDEL	SN	Up	Up	ND
		Striatum	NS	NS	No
	Climp-63	Striatum	NS	NS	No
Tubular ER shaping	RTN3	SN	NS	NS	ND
		Striatum	NS	NS	Yes
	RTN4	Striatum	NS	NS	No
	ATL3	Striatum	NS	NS	No
	REEP2	SN	Up	Up	ND
		Striatum	NS	NS	Yes
	REEP5	SN	Up	Up	ND
		Striatum	NS	NS	Yes
Phospholipid metabolism	Lpcat1	SN	Up	Up	ND
		Striatum	Up	Up	Yes

“ND”: Not detected; “NS”: No significant alteration in expression; “Up”: Up-regulated significantly; “Down”: Down-regulated significantly

The axonal enlargements largely appear in the NAc and are significantly more numerous per area than those in the CPu of *WDR45*^cKO^ mice (Fig. 2a, d, e). Interestingly, in our previously reported *VMP1*^*cKO*^ mouse model with conditional knockout of essential autophagic gene *VMP1* in mature DAergic neurons, more axonal enlargements were observed in the CPu than those in the NAc region [32]. The functional heterogeneity of midbrain DAergic neuron subtypes might lead to their differential vulnerability during the development stage and adulthood [50–52]. Additionally, the gene's function and distribution may also affect the cellular process that maintains DAergic neuron subtype characteristics. The underlying mechanisms for this pathological differential of axonal enlargements in various brain regions remain to be further investigated.

As an autophagic protein, WDR45 deficiency may cause autophagic damage and subsequently lead to DAergic neurodegeneration [3]. Nevertheless, *WDR45* is not an essential autophagic gene, as a mild autophagy defect was observed in *WDR45* knockout cell models and mouse models. All *WDR45*-deficit mice are born normally and survive in the postnatal period [4, 53], which is in contrast to those model mice lacking essential ATG proteins, like ATG5, ATG7, and VMP1 [32, 54, 55]. The autophagic substrates p62 and Ub were found to increase significantly in DAergic neurons of young *WDR45*^*cKO*^ mice (Fig. 3), supporting that autophagic inhibition was an early event. Following the disruption of autophagy, mitochondrial and RER-associated functions were disturbed [56, 57]. The expressions of most detected proteins associated with mitochondria and RER were dramatically altered in the DAergic neuronal soma of young *WDR45*^*cKO*^ mice (Fig. S4, Table 3). Such cumulative effects in the functional disturbance will develop into structural injury; on the other hand, it may further promote neurodegeneration [4]. Mitochondrial and RER turnover will be inhibited under an interrupted autophagic flux [56, 58]. A large number of damaged mitochondria and swollen RER tubules accumulated in the neuronal cytosol of aged *WDR45*^*cKO*^ mice (Fig. 1), which severely disrupted the cellular metabolic homeostasis [59]. Before the occurrence of structural damage, mitochondria undergo the hyperactivity of fusion processes mainly at a young age. Enhanced mitochondrial fusion activity promotes mitochondrial quality in response to energy demand, as longer and more interconnected (fused) mitochondria generated by fusion correlate with high respiration efficiency [60]. On the other way, mitochondrial fission was also highly activated from a young age and continues until the aged stage, evidenced by the upregulation of FIS1 expression in the DAergic neurons of both young and aged *WDR45*^*cKO*^ mice. Mitochondrial fission plays an important role in the removal of damaged mitochondria by autophagy [60]. The enhanced mitochondrial fission in the *WDR45*^*cKO*^ mice may imply an increase in the damaged mitochondria and induction of autophagy. Moreover, the apoptotic programs may also be activated by mitochondrial fission with aging [61].

The regulation of the energy metabolism by autophagy cannot be ignored in neurodegeneration, as autophagy itself is a catabolic mechanism that allows cells to deliver cytoplasmic contents to lysosomes for degradation to maintain cellular energy homeostasis and protect cells against stress [62]. Neurons are more sensitive to endogenous and exogenous stimuli, especially energy shortages. Mitochondria supply the energy in neurons through oxidative phosphorylation reactions [63]. In *WDR45*^*cKO*^ mice, mitochondrial quality control was severely disturbed, which will limit the energy supply for DAergic neurons. The striatum, a brain region with high density of DAergic synapses, has high energy demands and Ca^2+^ buffering requirements [63]. Given the anatomical structure of DAergic neurons, the distal axons, typically axonal terminals, are more prone to energy depletion [64]. This hypothesis was further proved by the subsequent proteomic analysis for striatal samples of *WDR45*^*cKO*^ mice. The metabolic processes were highly activated even at young ages (Fig. 5), verifying hyperactive metabolism and the underlying energy shortage in the striatum. Mitochondria in axons are shorter and ideally anchored at distal axons and synapses as local energy providers [65, 66]. Regulating the trafficking and anchoring status of axonal mitochondria ensures that metabolic areas are constantly supplied with ATP. Several pathways and molecules are crucial in regulating mitochondrial transport to meet axonal energy supply and facilitating axonal regeneration, such as AMP-activated protein kinase-p21-activated kinase energy signaling pathway, myosin VI [67], AKT-P21-activated kinase 5 axis [68], and syntaphilin [69]. The DEPs associated with mitochondrial function analyzed by our proteomic data, like succinate-CoA ligase GDP-forming subunit beta (Suclg2) and cytochrome C oxidase subunit 4I2 (Cox4i2) (Supplementary Table 1), function in the TCA cycle and the mitochondrial respiratory chain, respectively [70–72], may provide a clue to study the mechanism of energy supply in striatal region, especially in the DAergic axons.

Recent studies have shown that autophagy plays a vital role in axonal degeneration, such as regulating mature DAergic axon terminal morphology [32, 73, 74]. However, the underlying mechanism of how autophagy regulates axonal morphology is still unclear. Autophagy regulates lipid metabolism and is involved in axonal degeneration [75, 76]. Phospholipid metabolism was highly activated in the striatal region of *WDR45*^*cKO*^ mice. The phospholipid metabolism-related protein Lpcat1, an important ER-resident enzyme that catalyzes PC biosynthesis [77], was identified through proteomic analysis. The subsequent study indicated that Lpcat1 was involved in the axonal degeneration in *WDR45*^*cKO*^ mice. Lpcat1 recently was reported to regulate α-synuclein (αSyn) pathology and cytotoxicity [78]. Suppression of Lpcat1 reduces αSyn accumulations and toxicity, while overexpression of Lpcat1 promotes phosphorylated S129 αSyn positive aggregation [78]. DPPC, a catalytic product of Lpcat1, similarly promotes neuronal αSyn pre-formed fibril-seeded aggregation, indicating increased Lpcat1 and DPPC both promote the accumulation of toxic proteins [78]. In the current study, PC metabolism was the most altered, and increased DPPC was found in the young *WDR45*^*cKO*^ mice and may also have similar lipotoxicity to axons. The pivotal roles of phospholipids, especially PC metabolism, in axonal degeneration were also pointed out in recent studies [79, 80].

It is worth further investigation whether PC metabolism affects the structural composition of axonal ER, as PC accounts for 60% of the ER structural composition. ER interconnects with phospholipids; it provides the primary site for phospholipid synthesis, particularly PC synthesis, and in turn, the metabolism of phospholipids plays roles in the morphology and composition of ER [16]. Lpcat1 and tubular ER-shaping proteins (RTN3, REEP2, and REEP5) all appear at the site of axonal enlargement in *WDR45*^*cKO*^ mice, implying the potential interaction between phospholipids and tubular ER. Through TEM analysis, we found the morphology of tubular ER in striatal axons is fragmented in *WDR45*^*cKO*^ mice. The tubular ER morphology could be regulated by the specific phospholipid or regulated molecules [24, 81], such as the spontaneous membrane bending induced by high concentrations of LPC [82]. Although current studies have emphasized that autophagy regulates axonal morphology by controlling axonal ER [21], there is currently no evidence directly pointing out that autophagy controls ER morphology by regulating phospholipids metabolism in axonal degeneration. Further clarifying the relationship in neurons among autophagy, phospholipids/corresponding molecules, and ER morphology, will significantly improve the mechanistic understanding of axonal degeneration in BPAN and many other neurodegenerative diseases.

It is necessary to point out that among all validated proteins (Table 3), only Lpcat1, RTN3, REEP2, and REEP5 were accumulated in the axonal swellings, providing an important clue that phospholipid metabolism and tubular ER potentially interact with each other in the axonal degeneration. Additionally, the proportion of increased expression of Lpcat1 (cKO vs. cWT = 2.73) was much higher than that of REEP2 (cKO vs. cWT = 1.74) and REEP5 (cKO vs. cWT = 1.32) in the soma of DAergic neurons from young mice, which highlights the important role of phospholipid in axonal degeneration processes, even in the maintaining of tubular ER shape. Sheet ER, which represents rough ER, does not participate in axonal pathology because it is mainly responsible for protein synthesis, processing, and sorting in the soma [83], as confirmed by our finding that the sheet ER markers, KDEL, and Climp-63, do not colocalize with axonal enlargements in *WDR45*^*cKO*^ mice (Fig. S6).

Iron deposit in the brain is considered to promote neuronal degeneration in patients with *WDR45* mutations [84, 85]. However, in aged *WDR45*^*cKO*^ mice, the iron deposits were not observed in the SN and striatum (Fig. S8), so as the whole-body *WDR45* knockout mice and *WDR45*^*NesCre*^ mice that conditionally knockout *WDR45* in neurons [53, 86], which may indicate that the effect of gene knockout is somewhat different from that of gene mutation.

## Conclusions

In conclusion, our study provides proteomic and lipidomic profiles and insights into the pathological mechanisms underlying WDR45 deficiency-induced axonal degeneration. Our findings suggest that this degeneration involves Lpcat1 and associated phospholipid metabolism. While this study provides a molecular basis for axonal degeneration in BPAN and other neurodegenerative diseases, further mechanistic investigations are necessary to fully understand the complex relationship among Lpcat1 and its related phospholipid metabolism, tubular ER, and autophagy in axonal degeneration. In addition, there is an urgent need to examine the metabolism within individual neuronal axons in vivo, which may facilitate the development of more effective strategies for preventing or reversing axonal degeneration in the context of WDR45 deficiency and related disorders.

### Supplementary Information

Supplementary Material 1.

Supplementary Material 2.

Supplementary Material 3.

## Data Availability

All data generated in this study are included in this published article. Raw datasets during the current study are available from the corresponding author upon request.

## Abbreviations

ATG5: Autophagy gene 5
ATL: Atlastin
Amt: Aminomethyltransferase
ATP: Adenosine triphosphate
AHexCer: Acylhexosylceramide
αSyn: αSynuclien
Abhd4: Abhydrolase domain containing 4, N-acyl phospholipase B
APOD: Apolipoprotein D
Agt: Angiotensinogen
Apbb1ip: Amyloid $\upbeta$-A4 precursor protein-binding family B member 1-interacting protein
αSyn: α-Synuclien
BPAN: $\upbeta$-Propeller protein-associated neurodegeneration
BNIP3: BCL2 interacting protein 3
BSN: Presynaptic cytomatrix protein bassoon
BPAN: β-Propeller protein-associated neurodegeneration
BP: Biological processes
Eno3: Beta-enolase3
Cox4i2: Cytochrome C oxidase subunit 4I2
CPu: Caudate putamen
CDP-DAG: Cytidine diphosphate-triacylglycerol
Cirbp: Cold inducible RNA binding protein
Cobl: Protein cordon-bleu
Cer: Ceramide
CAR: Acylcarnitine
CL: Cardiolipin
CC: Cellular components
DAergic: Dopaminergic
DEPs: Differentially expressed proteins
DRD1: Dopamine receptor D1
DRD2: Dopamine receptor D2
DPPC: Dipalmitoyl-phosphatidylcholine
ER: Endoplasmic reticulum
Etnppl: Ethanolamine-phosphate phospho-lyase
Elac1: ElaC ribonuclease Z1
Eno3: Enolase 3
FA: Fatty acid
FIS1: Mitochondrial fission 1 protein
GO: Gene ontology
HexCer: Hexosyl ceramide
HOMER1: Homer scaffold protein 1
Ints4: Integrator complex subunit 4
IFC: Immunofluorescence
KDEL: Lysine-aspartic acid-glutamic acid-leucine
Krt2: Keratin 2
DAergic: Midbrain dopaminergic
LPC: Lysophosphatidylcholine
Ldhd: Lactate dehydrogenase D
Lpcat1: Lysophosphatidylcholine acyltransferase 1
Man1a1: Mannosyl-oligosaccharide 1,2-alpha-mannosidase IA
MFN1: Mitofusin 1
MGDG: Monogalactosyl diglyceride
MLKL: Mixed lineage kinase-like
MBP: Myelin basic protein
MF: Molecular functions
Man1a1: Mannosidase alpha class 1A member 1
NAc: Nucleus accumbens
NMNAT3: Nicotinamide nucleotide adenylyltransferase 3
OPA1: OPA1 mitochondrial dynamin like GTPase
Ophn1: Oligophrenin 1
PG: Phosphatidylglycerol
PS: Phosphatidylserine
PC: Phosphatidylcholine
PE: Phosphatidylethanolamine
PSD: Postsynaptic density
PIS: Phosphatidylinositol synthase
PCA: Principal component analysis
Phyhd1: Phytanoyl-CoA dioxygenase domain-containing protein 1
PPC: Palmitoyl-lysophosphatidylcholine
Prkra: Protein activator of interferon induced protein kinase EIF2AK2
RIPK3: Receptor-interacting protein kinase-3
RTN: Reticulon
REEP: Receptor accessory protein
SM: Sphingomyelin
SNc: Substantia nigra pars compacta
SN: Substantia nigra
SYT1: Synaptotagmin 1
Sparc: Secreted protein acidic and cysteine rich
Sbno2: Strawberry notch homolog 2
Snx32: Sorting nexin 32
TAM: Tamoxifen
TH: Tyrosine hydroxylase
TOM20: Translocase of outer mitochondrial membrane 20
Tiam: TIAM Rac1 Associated GEF 2
Suclg2: Succinate-CoA ligase GDP-forming subunit beta
SHexCer: Sulfatide HexCer
SYN1: Synapsin-1
Ub: Ubiquitin
vMAT2: Vesicular monoamine transporter member 2
VTA: Ventral tegmental area
WDR45: WD repeat domain 45
