# Supplementary material for: Pathological characteristics of axons and alterations of proteomic and lipidomic profiles in midbrain dopaminergic neurodegeneration induced by WDR45-deficiency
Source: Mol Neurodegener. 2024 Aug 26;19:62. doi: 10.1186/s13024-024-00746-4 (PMC11346282; doi:10.1186/s13024-024-00746-4)
Supplement: Supplementary file 1 — Supplementary Material 1. [file 13024_2024_746_MOESM1_ESM.docx]

**Supplementary**

Figure S1

Figure S2

Figure S3

Figure S4

Figure S5


Figure S6

Figure S7


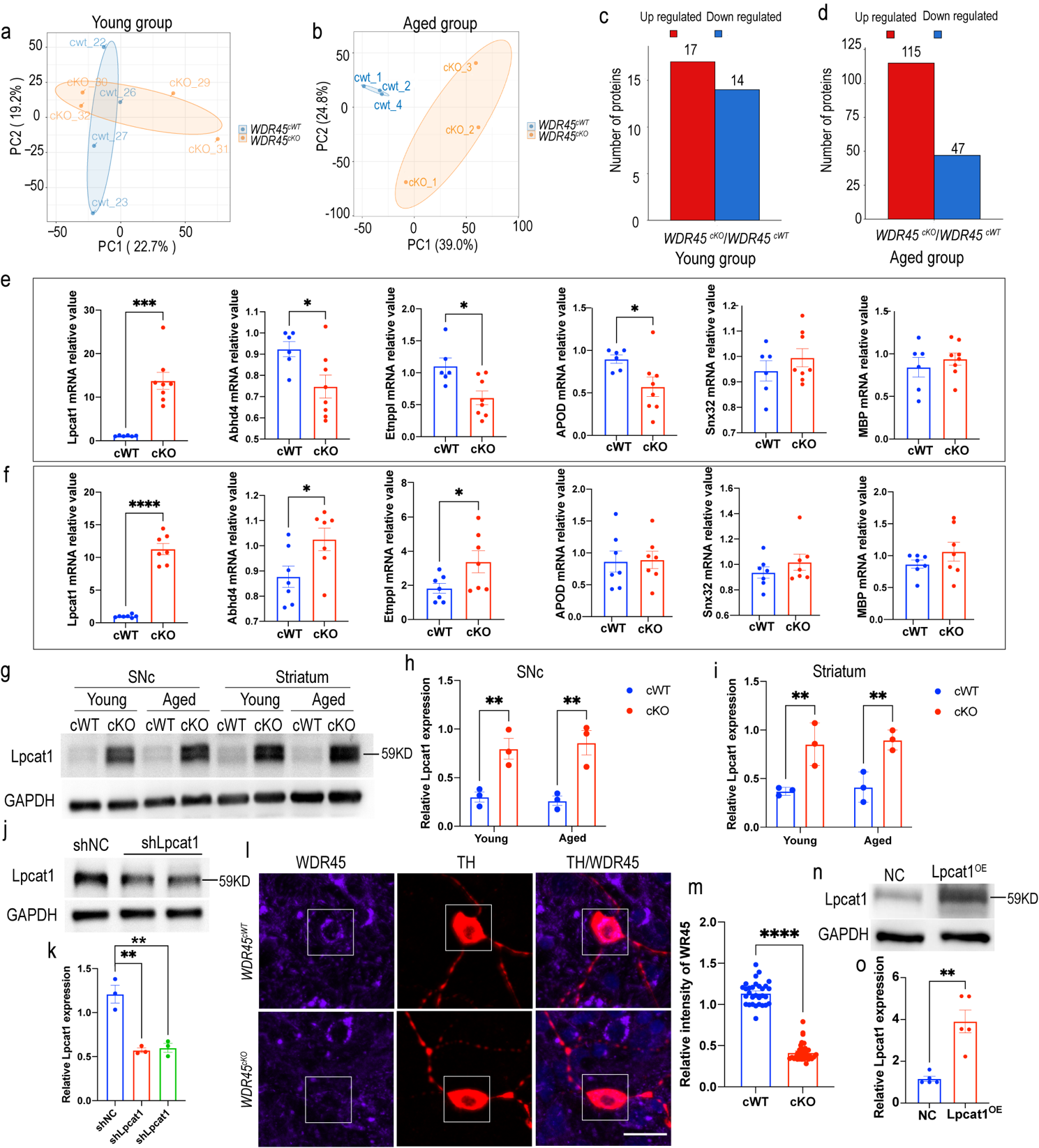


Figure S8

**Fig. S9**

**Supplementary figure legends**

**Figure S1 Conditional knockout of WDR45 in DAergic neurons.** **a** The basic strategy for generating TAM-inducible Cre/loxp-directed WDR45 knockout mice. A timeline for TAM administration and tissue collection is also presented. **b** PCR detection of the Cre transgene (upper) and WDR45 floxed allele (lower). IFC staining for WDR45 expression in DAergic neurons was performed using antibodies against WDR45 (green) and TH (red) in 6-month-old *WDR45^cWT^* and *WDR45^cKO^* mice **(c)** and the analysis for the proportion of TH- and WDR45-positive neurons(**d**). Scale bar: 250 μm. For high-magnification images: 10 μm. Data were analyzed using Student’s t-test. Data are represented as the mean±SEM. ^***^*p <* 0.001.

**Figure S2 The behavioral tests were performed in *WDR45^cWT^* mice and *WDR45^cKO^* mice. a-b** Total traveled distance and stereotypic counts of the young (6-8 months old), middle-aged (11-13 months old), and aged (17-19 months old) mice in the whole open field are presented (N=10-18 mice per genotype). **c** The latency to fall from the rotarod was recorded (N= 10-20 mice per genotype). **d** The mice that entered the a, b, or c arm were recorded, and the Y-maze alteration rate was calculated (N=10-15 mice per genotype). **e-h** Social approach and preference for the social novelty of middle-aged and aged *WDR45^cWT^* mice and *WDR45^cKO^* mice were examined in the 3-chamber social test (N=9-10 mice per genotype). The exploring time spent in social approach (**i**) and preference in social novelty (**j**). Data were analyzed using two-way ANOVA followed by Sidak’s multiple comparisons tests. Data are represented as the mean±SEM. ^*^*p* < 0.05, ^***^*p* < 0.001.

**Figure S3 The analysis for ER-mitochondria contacts and mitochondria damage in the striatum. a** Representative TEM images of observed mitochondria that contact with ER membrane in aged *WDR45^cWT^* mice and *WDR45^cKO^* mice. Scale bar, 500 nm. **b** Quantification of the mitochondria that contact with ER membrane in DAergic neurons (N= 15 slices from 3 mice per genotype). IFC staining of BNIP3 (green) with TH (red) in the striatum of young and aged *WDR45^cWT^* mice and *WDR45^cKO^* mice (**c)** and the analysis for the relative density of BNIP3 (**d, e)** (N=9 silces collected from young *WDR45^cWT^* mice and 9 slices from young *WDR45^cKO^* mice. N=14 silces collected from aged *WDR45^cWT^* mice and 11 slices from aged *WDR45^cKO^* mice). The nuclei were labeled with DAPI (blue). Scale bar, 20 μm. The analysis for the number of mitochondria collected from each TEM image of the striatum **(f)** and proportion of mitochondria with damaged cristae **(g)**. Data were analyzed using Student’s t-test. Data are represented as the mean±SEM. ^**^*p <* 0.01, ^***^*p <* 0.001.

**Figure S4 IFC staining for proteins associated with mitochondria and ER.** Double-label immunofluorescence of TOM20 (green) with TH (red) **a** and analysis for the relative fluorescence density of TOM20 in TH-positive neurons in the SNc of young (**a-1**) and aged (**a-2**) *WDR45^cWT^* mice and *WDR45^cKO^* mice (N=95 DAergic neurons collected from young *WDR45^cWT^* mice, 80 neurons from young *WDR45^cKO^* mice, 144 neurons collected from aged *WDR45^cWT^* mice, 233 neurons from aged *WDR45^cKO^* mice). The nuclei were labeled with DAPI (blue). Scale bar, 10 μm. **b** Double-label immunofluorescence of BNIP3 (green) with TH (red) and analysis for the relative fluorescence density of BNIP3 in TH-positive neurons in the SNc of young (**b-1**) and aged (**b-2**) *WDR45^cWT^* mice and *WDR45^cKO^* mice (N=96 DAergic neurons collected from young *WDR45^cWT^* mice and 178 neurons from young *WDR45^cKO^* mice, 183 neurons collected from aged *WDR45^cWT^* mice and 289 neurons from aged *WDR45^cKO^* mice). **c** Double-label immunofluorescence of FIS1 (green) with TH (red) and analysis for the relative fluorescence density of FIS1 in TH-positive neurons in the SNc of young (**c-1**) aged (**c-2)** *WDR45^cWT^* mice and *WDR45^cKO^* mice (N=163 DAergic neurons collected from young *WDR45^cWT^* mice and 252 neurons from young *WDR45^cKO^* mice, 184 neurons collected from aged *WDR45^cWT^* mice and 239 neurons from aged *WDR45^cKO^* mice). **d** Double-label immunofluorescence of MFN1 (green) with TH (red) and analysis for the relative fluorescence density of MFN1 in TH-positive neurons in the SNc of young (**d-1**) and aged (**d-2**) *WDR45^cWT^* mice and *WDR45^cKO^* mice (N=52 DAergic neurons collected from young *WDR45^cWT^* mice and 103 neurons from age-matched *WDR45^cKO^* mice, 241 neurons collected from aged *WDR45^cWT^* mice and 231 neurons from age-matched *WDR45^cKO^* mice). **e** Double-label immunofluorescence of OPA1 (green) with TH (red) and analysis for the relative fluorescence density of OPA1 in TH-positive neurons in the SNc of young (**e-1**) and aged (**e-2**) *WDR45^cWT^* mice and *WDR45^cKO^* mice (N=71 DAergic neurons collected from young *WDR45^cWT^* mice and 134 neurons from young *WDR45^cKO^* mice, 167 neurons collected from aged *WDR45^cWT^* mice and 190 neurons from age-matched *WDR45^cKO^* mice). **f** Double-label immunofluorescence of KDEL (green) with TH (red) and analysis for the relative fluorescence density of KDEL in TH-positive neurons in the SNc of young (**f-1**) and aged (**f-2**) *WDR45^cWT^* mice and *WDR45^cKO^* mice (N=105 DAergic neurons collected from young *WDR45^cWT^* mice and 101 neurons from age-matched *WDR45^cKO^* mice, 136 neurons collected from aged *WDR45^cWT^* mice and 121 neurons from age-matched *WDR45^cKO^* mice). **g** Double-label immunofluorescence of SEC16A (green) with TH (red) and analysis for the relative fluorescence density of SEC16A in TH-positive neurons in the SNc of young (**g-1**) and aged (**g-2**) *WDR45^cWT^* mice and *WDR45^cKO^* mice (N=123 neurons collected from young *WDR45^cWT^* mice and 173 neurons from young *WDR45^cKO^* mice, 166 neurons collected from aged *WDR45^cWT^* mice and 124 neurons from age-matched *WDR45^cKO^* mice). **h** Double-label immunofluorescence of SEC31A (green) with TH (red) and analysis for the relative fluorescence density of SEC31A in TH-positive neurons in the SNc of young (**h-1**) and aged (**h-2**) *WDR45^cWT^* mice and *WDR45^cKO^* mice (N=74 DAergic neurons collected from young *WDR45^cWT^* mice and 82 neurons from age-matched *WDR45^cKO^* mice, 128 neurons collected from aged *WDR45^cWT^* mice and 204 neurons from aged *WDR45^cKO^* mice). **i** Double-label immunofluorescence of RTN3 (green) with TH (red) and analysis for the relative fluorescence density of RTN3 in TH-positive neurons in the SNc of young (**i-1**) and aged (**i-2**) *WDR45^cWT^* mice and *WDR45^cKO^* mice (N=62 DAergic neurons collected from young *WDR45^cWT^* mice and 93 neurons from age-matched *WDR45^cKO^* mice, 85 neurons collected from aged *WDR45^cWT^* mice and 154 neurons from aged *WDR45^cKO^* mice). **j** Double-label immunofluorescence of REEP2 (green) with TH (red) and analysis for the relative fluorescence density of REEP2 in TH-positive neurons in the SNc of young (**j-1**) and aged (**j-2**) *WDR45^cWT^* mice and *WDR45^cKO^* mice (N=47 DAergic neurons collected from young *WDR45^cWT^* mice and 57 neurons from age-matched *WDR45^cKO^* mice, 118 neurons collected from aged *WDR45^cWT^* mice and 114 neurons from aged *WDR45^cKO^* mice). **k** Double-label immunofluorescence of REEP5 (green) with TH (red) and analysis for the relative fluorescence density of REEP5 in TH-positive neurons in the SNc of young (**k-1**) and aged (**k-2**) *WDR45^cWT^* mice and *WDR45^cKO^* mice (N=57 DAergic neurons collected from young *WDR45^cWT^* mice and 53 neurons from age-matched *WDR45^cKO^* mice, 71 neurons collected from aged *WDR45^cWT^* mice and 142 neurons from aged *WDR45^cKO^* mice). The nuclei were labeled with DAPI (blue). Scale bar, 10 μm. Data were analyzed using Student’s t-test. Data are represented as the mean±SEM. ^****^*p <* 0.0001, ^***^*p <* 0.001, ^*^*p <* 0.05.

**Figure S5 The detections for DAergic-related receptors and synaptic proteins in the striatum by IFC staining. a** Immunofluorescence of DAT (red) with TH (green) in the striatum of young and aged *WDR45^cWT^* mice and *WDR45^cKO^* mice the analysis for the relative density of DAT (**a-1, a-2)** (N=6 slices collected from young *WDR45^cWT^* mice and 6 slices from age-matched *WDR45^cKO^* mice, 17 slices collected from aged *WDR45^cWT^* mice and 11 slices from aged *WDR45^cKO^* mice). **b** Co-staining of DRD1 (green) with TH (red) in the striatum of young and aged *WDR45^cWT^* mice and *WDR45^cKO^* mice and the analysis for the relative density of DRD1 (**b-1, b-2)** (N=9 slices collected from young *WDR45^cWT^* mice and 10 slices from young *WDR45^cKO^* mice, 9 slices collected from aged *WDR45^cWT^* mice and 11 slices from aged *WDR45^cKO^* mice). **c** IFC staining of DRD2 (green) with TH (red) in the striatum of aged *WDR45^cWT^* mice and *WDR45^cKO^* mice and the analysis for the relative density of DRD2 (**c-1, c-2)** (N=10 slices collected from young *WDR45^cWT^* mice and 10 slices from young *WDR45^cKO^* mice, 13 slices collected from aged *WDR45^cWT^* mice and 14 slices from aged *WDR45^cKO^* mice). **d** Co-staining of vMAT2 (green) with TH (red) in the striatum of aged *WDR45^cWT^* mice and *WDR45^cKO^* mice and the analysis for the relative density of vMAT2 (**d-1, d-2)** (N=7 slices collected from young *WDR45^cWT^* mice and 7 slices from young *WDR45^cKO^* mice, 17 slices collected from aged *WDR45^cWT^* mice and 13 slices from aged *WDR45^cKO^* mice). **e** IFC analysis of synapse-related proteins in the striatum of young *WDR45^cWT^* mice and age-matched *WDR45^cKO^* mice. Scale bar, 20 μm. **e-1** Quantifying PSD95' fluorescence density (N=8 slices from 3 mice per genotype). **e-2** Quantifying SYT1' fluorescence density (N=8 slices from 3 mice per genotype). **e-3** Quantifying SYN1' fluorescence density (N=8 slices from 3 mice per genotype). **e-4** Quantifying HOMER1' fluorescence density (N=10 slices from 3 mice per genotype). **e-5** Quantifying BSN' fluorescence density (N=7 slices from 3 mice per genotype). The nuclei were labeled with DAPI (blue). Scale bar, 20 μm. Data were analyzed using Student’s t-test. Data are represented as the mean±SEM. ^****^*p <* 0.0001, ^*^*p <* 0.05.

**Figure S6 Co-localization of ER markers and DAergic axonal swellings in the striatum. a** Double-label immunofluorescence of RTN4 (green) with TH (red) in the striatum of aged *WDR45^cWT^* mice and *WDR45^cKO^* mice, and analysis for colocalization of RTN4 vs TH by using three parametrers: Pearson's R value (above threshold), Manders' tM1 (Above autothreshold of Ch2), and Manders' tM2 (Above autothreshold of Ch1 (**b**). Approaching 0 indicates weak correlation. **c** Double-label immunofluorescence of KDEL (green) with TH (red) in the striatum of aged *WDR45^cWT^* mice and *WDR45^cKO^* mice, and analysis for colocalization of KDEL vs. TH (**d**). **e** Double-label immunofluorescence of Climp-63 (green) with TH (red) in the striatum of aged *WDR45^cWT^* mice and *WDR45^cKO^* mice, and analysis for colocalization of Climp-63 vs. TH (**f**). **g** Double-label immunofluorescence of ATL3 (green) with TH (red) in the striatum of aged *WDR45^cWT^* mice and *WDR45^cKO^* mice, and analysis for colocalization of ATL3 vs. TH (**h**). The nuclei were labeled with DAPI (blue). Scale bar, 10 μm.

**Figure S7 The qRT-PCR analysis for the expressions of genes encoding DEPs of regulation in lipid metabolism.** PCA score plot of the striatal proteome of young (**a)** and aged (**b**) *WDR45^cWT^* and *WDR45^cKO^* mice. The statistical analysis for DEPs (fold change >1.3, *p <* 0.05) from both young (**c**) and aged (**d**) mice. **e** qRT-PCR analysis for the mRNA expressions of target genes from the striatum of young *WDR45^cWT^* and *WDR45^cKO^* mice (n=6-8 mice per genotype). **f** qRT-PCR analysis for the mRNA expressions of target genes from the striatum of aged *WDR45^cWT^* and *WDR45^cKO^* mice (n=7-8 mice per genotype). Representative WB image (**g**) and analysis for Lpcat1 expression in SNc (N=3 mice per genotype) (**h**) and striatum (N=3 mice per genotype) (**i**). Representative WB image (**j**) and analysis (**k**) for Lpcat1 expression in primary midbrain neuron culture with LV-shLpcat1 (or LV-shNC) transfection collected at DIV13 (N=3 independent repeated experiments). The examination of WDR45 expression (pseudo-color) in DAergic neurons (red) from primary midbrain neuron culture with 4-OHT treatment by IFC staining (**l**) and analysis for WDR45 relative density in each DAergic neurons (N=30 primary DAergic neurons from *WDR45^cWT^* and 33 primary DAergic neurons from *WDR45^cKO^* mice) (**m**). Scale bar: 20 μm. Representative WB image (**n**) and analysis (**o**) for Lpcat1 expression in primary midbrain neuron cultures with LV-Lpcat1 (or LV-NC) transfection collected at DIV11 (N=5 independent repeated experiments). Data were analyzed using Student’s t-test (e, f, k, m, o) and two-way ANOVA followed by Sidak’s multiple comparisons tests (h, i). Data are represented as the mean±SEM. ^****^*p <* 0.0001, ^***^*p <* 0.001, ^**^*p <* 0.01, ^*^*p <* 0.05.

**Fig S8 Iron staining by using the Prussian blue stain method.** Representative immunochemical staining images of iron. A large blue deposit will be seen if iron is accumulated in brain sections. Scale bar, 50 μm for SNc; 100 μm for striatum.

**Fig. S9 NMNAT3 expression in the striatum and SN.** IFC staining of NMNAT3 (pseudo color) with TH (red) in the SN (**a**) and striatum (**b**) of young *WDR45^cWT^* mice and *WDR45^cKO^* mice. Scale bar: 10 μm. IFC of NMNAT3 (pseudo color) with TH (red) in the SN (**c**) and striatum (**d**) of aged *WDR45^cWT^* mice and *WDR45^cKO^* mice. Scale bar: 10 μm. Representative WB image of NMNAT3 (**e**) and analysis (**f, g**) for NMNAT3 protein expression in the striatum and SNc (N=3 independent repeated experiments). **h** The protein expression of NMNAT3 in the striatum from proteomic analysis. Data (f-h) were analyzed using two-way ANOVA followed by Sidak’s multiple comparisons tests. Data are represented as the mean±SEM. ^****^*p <* 0.0001, ^***^*p <* 0.001, ^**^*p <* 0.01, ^*^*p <* 0.05.
